# Future time perspective and training procrastination in Chinese collegiate athletes: self-control mediation and the roles of mastery-approach and performance-avoidance goals

**DOI:** 10.3389/fpsyg.2026.1873288

**Published:** 2026-07-15

**Authors:** Jinrui Zhang

**Affiliations:** School of Sports Training, Chengdu Sport University, Chengdu, China

**Keywords:** Chinese collegiate athletes, future time perspective, mastery-approach goal orientation, performance-avoidance goal orientation, self-control, training procrastination

## Abstract

**Purpose:**

This cross-sectional study examined the association between future time perspective (FTP) and training procrastination in Chinese collegiate athletes, with self-control as a mediator and mastery-approach (MAp) and performance-avoidance (PAv) goals as motivational boundary conditions.

**Methods:**

Participants were 1,674 collegiate athletes from 48 Chinese higher education institutions in 15 provincial-level regions. FTP was measured with the Future subscale of the Zimbardo Time Perspective Inventory, self-control with the Brief Self-Control Scale, MAp and PAv goals with selected subscales of the Achievement Goal Questionnaire for Sport, and training procrastination with a training-adapted Irrational Procrastination Scale. Confirmatory factor analysis evaluated measurement validity. Structural equation modeling tested the indirect pathway through self-control. Hierarchical regression and PROCESS Model 5 tested direct and separate moderation effects, and simultaneous-interaction regression entered both goal orientations and interactions as a robustness check.

**Results:**

FTP was negatively associated with training procrastination (β = −0.342, *p* < 0.001) and positively associated with self-control (β = 0.211, *p* < 0.001); self-control was negatively associated with training procrastination (β = −0.295, *p* < 0.001). The indirect effect via self-control was significant (β = −0.063, 95% CI [−0.080, −0.046]), accounting for 15.56% of the total effect. In separate PROCESS models, MAp strengthened the negative FTP–procrastination association (*B* = −0.072, *p* < 0.001), whereas PAv weakened it (*B* = 0.058, *p* < 0.01). In the simultaneous-interaction model, FTP × MAp remained significant (*B* = −0.058, *p* = 0.020), whereas FTP × PAv was non-significant (*B* = 0.028, *p* = 0.272).

**Conclusion:**

FTP was associated with lower training procrastination partly through self-control. MAp emerged as a robust motivational boundary condition, whereas the independent moderating role of PAv was less robust after accounting for MAp. Findings suggest that training procrastination is associated with athletes’ future-oriented thinking, self-regulatory capacity, and motivational meaning attached to training. Results may inform coach education and athlete-support practices that connect daily training with future goals, support self-regulation, and encourage mastery-oriented feedback. Longitudinal and intervention studies are needed to clarify causal and developmental processes. These cross-sectional self-report findings do not establish causal relationships or directly assess broader sustainable athlete development outcomes.

## 1 Introduction

### 1.1 Sustainable athlete development and training procrastination among Chinese collegiate athletes

Sustainable athlete development has become an increasingly important concern in sport psychology, talent development, and health-related sport sciences (Serpa, 2025). Rather than viewing athletic success solely through short-term performance outcomes, recent perspectives emphasize the need to understand athlete development as a long-term, multidimensional process that integrates performance, psychological wellbeing, physical health, education, identity development, and continued sport participation (Miller and Kerr, 2002; Bergeron et al., 2015). From this perspective, sustainable development does not simply mean producing athletes who can perform well in the present. It also means helping athletes maintain adaptive motivation, effective self-regulation, psychological resilience, and healthy engagement across different stages of their athletic pathway (Kuchar et al., 2023; Tóth et al., 2024).

This issue is particularly relevant for collegiate athletes. Unlike full-time professional athletes, collegiate athletes often need to manage a dual role as both students and athletes. They are expected to complete academic requirements while also participating in systematic training, competitions, team activities, and performance evaluations (Aycocho et al., 2024). This dual-role context may provide opportunities for holistic development, but it also creates considerable self-regulatory demands (Koole et al., 2012). Athletes must decide how to allocate time and effort, how to cope with fatigue and pressure, and how to maintain training engagement when academic, social, and personal demands compete for attention (Macquet and Skalej, 2015). For Chinese collegiate athletes, these challenges may be especially salient because athletic development is often embedded within educational institutions, where sport performance and academic progression are both valued (Lyu et al., 2022). Therefore, understanding the psychological factors associated with effective training behavior among Chinese collegiate athletes is important for promoting both performance and wellbeing.

One behavioral pattern that may interfere with consistent training engagement is training procrastination. Procrastination is generally understood as the voluntary delay of intended action despite expecting that the delay may lead to negative consequences (Steel, 2007). In the context of sport training, training procrastination may involve delaying planned exercises, avoiding difficult or unpleasant training tasks, postponing preparation or recovery routines, or failing to initiate training-related behaviors in a timely manner (Kasper, 2019). Although procrastination has been widely studied in academic, occupational, and general life domains, its implications for athletic training remain comparatively underexplored. This is a notable gap because training is inherently future-oriented. The benefits of training rarely appear immediately; they are accumulated through repeated effort, deliberate practice, and long-term commitment. When athletes procrastinate in training, they may not only reduce training quality in the short term, but also weaken the consistency of long-term development (Fuke et al., 2022).

Training procrastination should therefore not be regarded merely as a problem of time management. It may reflect a deeper difficulty in translating long-term athletic goals into present action. In this sense, training procrastination is closely connected with self-regulation, motivation, and sustained sport participation. Athletes who frequently delay training-related tasks may experience lower perceived competence, greater psychological pressure, reduced confidence, and unstable performance development. Over time, such patterns may be associated with both athletic progress and psychological wellbeing. Identifying the psychological antecedents and mechanisms of training procrastination among Chinese collegiate athletes can therefore provide useful evidence for athlete-support practices that promote healthier and more consistent training engagement (Ferrari et al., 1995).

In the present study, sustainable athlete development is used as a contextual and interpretive framework rather than as a directly measured construct. The empirical focus is on training procrastination as a behavioral tendency that may be relevant to training consistency. Therefore, the findings are interpreted as informing sustainable training engagement, but they should not be taken as direct evidence of broader athlete sustainability outcomes.

### 1.2 Future time perspective and training procrastination

Future time perspective is one psychological factor that may be closely associated with training procrastination. Future time perspective refers to the tendency to think about, value, and organize present behavior in relation to future goals and consequences (Zimbardo and Boyd, 1999). Individuals with a stronger future time perspective are more likely to plan ahead, delay immediate gratification, consider long-term outcomes, and regulate current behavior in accordance with future aspirations (Pang et al., 2014). In achievement contexts, future-oriented individuals are generally more capable of linking present effort with later success, which may help them persist in goal-directed activities even when immediate rewards are limited (Göllner et al., 2018).

Sport training provides a meaningful context in which to examine the role of future time perspective, because prior sport motivation research has linked goal distance in time and future time orientation with achievement motives and performance-related indicators in sports (Thomassen et al., 2001). Stolarski et al. (2019) proposed a sport-specific temporal perspective framework in which athletes’ time perspectives may shape sport functioning and performance through multiple pathways, including sport motivation and engagement, affective regulation, and appraisal of performance and coping with related emotions. This framework is particularly relevant to training procrastination because athletic training requires athletes to connect present effort with delayed performance outcomes, regulate discomfort during demanding practice, and interpret setbacks as part of long-term development rather than as reasons for avoidance. In this sense, future time perspective is not only a general cognitive orientation toward time; in sport settings, it may shape how athletes evaluate the meaning of effortful training tasks and how they respond to the delayed rewards of athletic improvement.

Athletic improvement often requires athletes to engage in repetitive, demanding, and sometimes monotonous training activities. The value of these activities may not be immediately visible, especially when athletes face fatigue, temporary performance setbacks, injury concerns, or competing academic responsibilities. Under such conditions, athletes with a stronger future time perspective may be more likely to interpret current training as an investment in future performance, health, and personal development. They may be better able to recognize that today’s training behavior contributes to future competitive readiness, technical improvement, and long-term athletic identity (Stolarski et al., 2026; Vansteenkiste et al., 2004).

By contrast, athletes with a weaker future time perspective may be more vulnerable to short-term preferences and immediate discomfort. When the future value of training is unclear or psychologically distant, athletes may be more likely to postpone difficult tasks, choose more immediately rewarding alternatives, or avoid training activities that require effort and persistence (Codina et al., 2024). In this way, low future orientation may be associated with a higher likelihood of training procrastination. For collegiate athletes, this process may be even more pronounced because training goals often compete with academic deadlines, social life, and other short-term demands (Gao et al., 2025). A stronger future time perspective may help athletes prioritize training-related behaviors by making long-term athletic development more psychologically meaningful (Stolarski et al., 2026).

Within the context of sustainable athlete development, future time perspective is important because it links present self-regulation with long-term growth (Vansteenkiste et al., 2004). Sustainable training engagement requires athletes to maintain effort over time rather than rely only on temporary motivation or external pressure (Nazarpouri et al., 2025). If athletes perceive training as part of a broader future-oriented pathway, they may be less likely to delay training-related tasks unnecessarily (Codina et al., 2024). Therefore, the present study proposes that future time perspective is negatively associated with training procrastination among Chinese collegiate athletes.

### 1.3 Self-control as a mediating mechanism

Although future time perspective may be associated with lower training procrastination, this relationship is unlikely to occur automatically. Future-oriented thinking needs to be translated into actual behavior through self-regulatory capacity. Self-control refers to the ability to regulate impulses, resist temptations, inhibit short-term distractions, and act in accordance with long-term goals (Tangney et al., 2004). Individuals with higher self-control are generally better able to persist in planned activities, manage competing desires, and maintain goal-directed behavior when immediate motivation is insufficient (Inzlicht et al., 2014).

Self-control is highly relevant to sport training. Athletic training often requires athletes to overcome immediate discomfort, fatigue, boredom, or emotional fluctuation. Even when athletes understand the long-term importance of training, they may still struggle to begin or complete training tasks if they lack sufficient self-control (Balk and Englert, 2020). For example, a collegiate athlete may know that consistent strength training contributes to future performance, but still delay the session because of tiredness, academic stress, or the attraction of leisure activities. In such situations, self-control helps athletes act in line with long-term athletic goals rather than immediate preferences.

Future time perspective may be positively associated with self-control because it increases the perceived value of long-term outcomes. When athletes clearly recognize the connection between present training and future development, they may become more willing to regulate short-term impulses (Blouin-Hudon and Pychyl, 2017). Future-oriented athletes are more likely to plan their behavior, resist distractions, and persist in difficult tasks because they understand why current effort matters. In this sense, future time perspective may provide a motivational basis for self-control, while self-control may provide the behavioral capacity through which future-oriented intentions are implemented (Miao et al., 2024).

Self-control may, in turn, be negatively associated with training procrastination. Procrastination is often described as a failure of self-regulation because individuals delay intended actions despite knowing that delay may be harmful (Steel, 2007). In training contexts, athletes with stronger self-control may be more capable of starting planned tasks on time, maintaining effort during challenging sessions, and following training routines even when immediate enjoyment is low. Conversely, athletes with lower self-control may be more likely to give in to avoidance, distraction, or short-term comfort, thereby increasing training procrastination.

Accordingly, self-control may represent one self-regulatory pathway through which future time perspective is associated with training procrastination. Future time perspective may be linked to lower training procrastination partly because it is associated with stronger self-control, which then helps athletes transform future-oriented goals into more consistent training behavior (Khoirunnisa et al., 2024; Mao et al., 2022). This mechanism is especially relevant to sustainable training engagement because it explains not only whether future-oriented athletes report less procrastination, but also one possible way in which future-oriented thinking is connected with training behavior. However, because future time perspective also reflects broader temporal meaning, goal clarity, and future value, self-control is unlikely to exhaust all mechanisms linking future time perspective to training procrastination. In the present cross-sectional study, this proposed ordering is tested as a statistical indirect pathway rather than as an established temporal mechanism.

### 1.4 Achievement goal orientations as motivational boundary conditions

Although future time perspective and self-control may explain important aspects of training procrastination, athletes do not pursue future goals in the same motivational way. Self-Determination Theory provides an additional lens for understanding the motivational quality of future-oriented training goals. From this perspective, athletes are more likely to sustain effort when training goals are internalized, competence-supportive, and connected with personal development rather than experienced only as externally controlled demands (Ryan and Deci, 2000). Therefore, future time perspective may be more adaptive when future goals are experienced as self-endorsed and mastery-oriented, whereas future concerns framed by external pressure or negative evaluation may be less closely associated with timely training behavior.

Achievement goal theory further explains how athletes define competence, success, and failure in achievement settings. The 2 × 2 achievement goal framework distinguishes four types of goal orientations: mastery-approach, mastery-avoidance, performance-approach, and performance-avoidance (Elliot and McGregor, 2001). In sport, this framework has been applied to examine how athletes’ goal orientations shape motivation, affect, persistence, and performance-related behavior (Conroy et al., 2003). In broader training and work contexts, goal orientations have also been linked to transfer of training and the longitudinal utilization of skills (Gegenfurtner et al., 2016; Klonek et al., 2023).

These four achievement goal orientations reflect qualitatively different motivational patterns. Mastery-approach goals involve striving to improve, learn, and perform as well as one possibly can. Mastery-avoidance goals involve concern about not mastering a task or failing to meet one’s own standards. Performance-approach goals involve striving to perform better than others, whereas performance-avoidance goals involve trying to avoid performing worse than others (Conroy et al., 2003; Elliot and McGregor, 2001). Importantly, these dimensions should not be treated as a single overall score. A high score on mastery-approach orientation does not have the same psychological meaning as a high score on performance-avoidance orientation. Therefore, achievement goal orientations are better understood as distinct motivational tendencies that may shape how athletes respond to future-oriented goals (Conroy and Elliot, 2004).

The present study focuses on mastery-approach and performance-avoidance goals because they represent a theoretically meaningful motivational contrast for the proposed model. Mastery-approach goals emphasize self-improvement, skill development, and controllable progress, which are compatible with future-oriented training investment. Performance-avoidance goals emphasize avoiding poor performance and negative evaluation, which may transform future concerns into threat-based motivation. Mastery-avoidance and performance-approach goals are also important dimensions within the 2 × 2 framework, but they were beyond the scope of the present model because their expected roles in training procrastination may be more complex and context-dependent. Thus, focusing on mastery-approach and performance-avoidance goals allows the present study to examine two contrasting motivational conditions under which future time perspective may be more or less strongly associated with training procrastination.

Mastery-approach orientation is closely aligned with sustainable training engagement because it emphasizes personal improvement, skill mastery, effort, and long-term growth (Howell and Watson, 2007). Athletes with strong mastery-approach goals are likely to view training as a meaningful opportunity to improve themselves rather than merely as an external requirement. For these athletes, future time perspective may be especially relevant to lower training procrastination because their future goals are connected with self-improvement and developmental progress (Gao et al., 2012; Xiang et al., 2022). When future-oriented thinking is combined with mastery-approach motivation, athletes may be more likely to interpret present training as valuable, initiate training tasks promptly, and persist in difficult activities.

Thus, mastery-approach orientation may strengthen the negative association between future time perspective and training procrastination (Schmidt et al., 2009). Among athletes with high mastery-approach orientation, future time perspective should be more strongly associated with lower training procrastination because these athletes are more likely to transform future goals into constructive training engagement. By contrast, among athletes with low mastery-approach orientation, future time perspective may still be beneficial, but its association with lower training procrastination may be weaker because future goals are less strongly connected with self-improvement and mastery (Diaconu-Gherasim et al., 2023).

Performance-avoidance orientation may operate in the opposite way. Athletes with strong performance-avoidance goals are primarily concerned with avoiding poor performance, negative evaluation, or being worse than others (Isoard-Gautheur et al., 2013). Although such athletes may also think about the future, their future-oriented thoughts may be accompanied by fear, pressure, and defensive concerns. In achievement contexts, avoidance-oriented motivation is often associated with anxiety, avoidance tendencies, and less adaptive engagement (Stoeber et al., 2009). In sport training, athletes high in performance-avoidance orientation may interpret future outcomes as threats rather than opportunities for growth. When training tasks are difficult or evaluative, they may delay action as a way to avoid discomfort, possible failure, or negative self-evaluation.

Therefore, performance-avoidance orientation may weaken the negative association between future time perspective and training procrastination (Phan, 2009). Even if athletes have a strong future orientation, a dominant concern with avoiding failure may interfere with the translation of future goals into timely training behavior (Li and Lv, 2022). Instead of supporting action, future-oriented concerns may increase pressure and avoidance when they are filtered through performance-avoidance goals. In this case, the association between future time perspective and lower training procrastination may become weaker.

### 1.5 The present study and hypotheses

The present study examines training procrastination among Chinese collegiate athletes within the context of sustainable athlete development. By integrating future time perspective, self-control, and achievement goal orientations, this study aims to clarify the psychological process through which future-oriented thinking is associated with training procrastination, as well as the motivational conditions under which this association becomes stronger or weaker. Specifically, the study proposes that future time perspective is negatively associated with training procrastination, and that self-control mediates this relationship (Zimbardo and Boyd, 1999; Steel, 2007). In addition, the study examines mastery-approach and performance-avoidance goal orientations as two theoretically meaningful boundary conditions. Mastery-approach orientation is expected to strengthen the negative association between future time perspective and training procrastination, whereas performance-avoidance orientation is expected to weaken this association (Phan, 2009).

This study contributes to the literature in several ways. First, it extends procrastination research into the sport training context by focusing on training procrastination rather than general or academic procrastination. Second, it addresses Chinese collegiate athletes, a population that faces simultaneous academic and athletic demands and is therefore especially relevant to discussions of sustained training engagement and athlete development. Third, by examining self-control as a mediator, the study explains one self-regulatory pathway through which future time perspective may be associated with lower training procrastination. Finally, by examining mastery-approach and performance-avoidance goal orientations as moderators, the study recognizes that the role of future-oriented thinking may depend on how athletes define success and failure in sport.

Based on the above theoretical reasoning, the following hypotheses are proposed:

*H1*: Future time perspective is negatively associated with training procrastination among Chinese collegiate athletes.

*H2*: Self-control mediates the relationship between future time perspective and training procrastination. Specifically, future time perspective is positively associated with self-control, and self-control is negatively associated with training procrastination.

*H3*: Mastery-approach goal orientation moderates the relationship between future time perspective and training procrastination. The negative relationship between future time perspective and training procrastination is stronger among athletes with higher mastery-approach orientation.

*H4*: Performance-avoidance goal orientation moderates the relationship between future time perspective and training procrastination. The negative relationship between future time perspective and training procrastination is weaker among athletes with higher performance-avoidance orientation.

**FIGURE 1:**
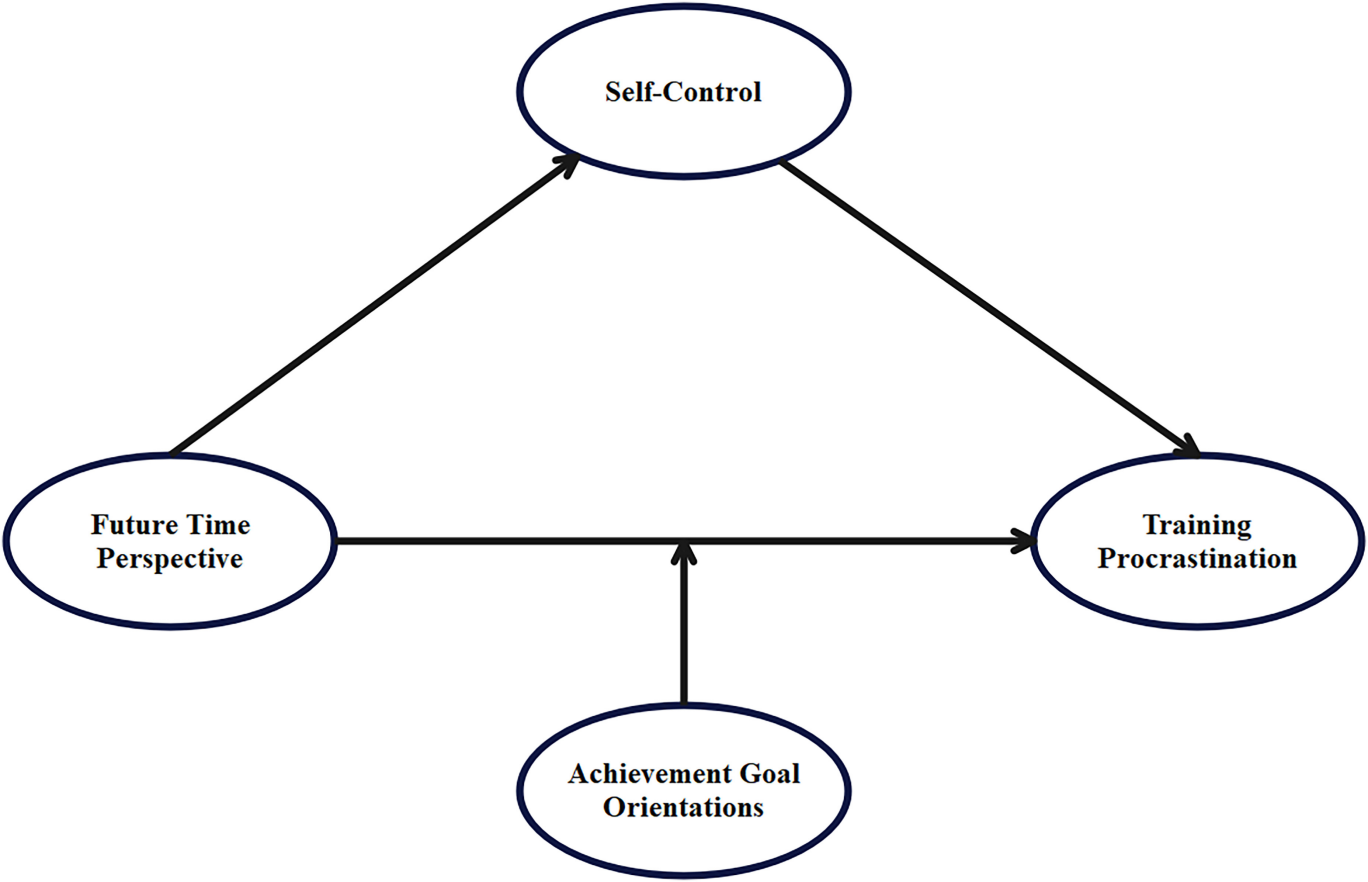
Theoretical model of the relationships among future time perspective, self-control, mastery-approach and performance-avoidance goal orientations, and training procrastination. Future time perspective is expected to be negatively associated with training procrastination and positively associated with self-control. Self-control is expected to be negatively associated with training procrastination. Mastery-approach and performance-avoidance goal orientations are expected to moderate the direct association between future time perspective and training procrastination.

Taken together, the proposed model suggests that future time perspective is associated with training procrastination both directly and indirectly through self-control, while mastery-approach and performance-avoidance goal orientations shape the strength of the direct association between future time perspective and training procrastination. A concise theoretical model of the present study is shown in Figure 1.

## 2 Materials and methods

### 2.1 Participants and procedure

This study adopted a cross-sectional survey design to examine the relationships among future time perspective, self-control, achievement goal orientations, and training procrastination among Chinese collegiate athletes. A multi-institutional convenience and network-based sampling strategy was used. Participants were recruited from 48 higher education institutions across 15 provincial-level administrative regions in China, including Guangdong, Henan, Shanghai, Sichuan, Anhui, Tianjin, Shandong, Chongqing, Jiangsu, Yunnan, Beijing, Guizhou, Zhejiang, Hebei, and Shanxi. Although the sample covered multiple universities and regions, it should not be interpreted as a probability sample of all Chinese collegiate athletes.

The target population consisted of adult collegiate athletes who were enrolled in higher education institutions and had current sport training experience. The inclusion criteria were as follows: participants had to be 18 years of age or older, currently enrolled as college students, self-report current engagement in systematic sport training as collegiate athletes, and be able to complete the questionnaire independently. Participants who did not meet these criteria were excluded from the final dataset. In the present study, regular sport training participation was based on participants’ self-reported current engagement in systematic sport training. Fixed eligibility thresholds for weekly training frequency or training duration were not used. Sport participation was recorded at the broader sport-type level, namely individual sport versus team sport; specific sport disciplines were not collected.

Ethical approval was obtained from the Ethics Committee of the School of Sports Training, Chengdu Sport University, China (Approval No. CTYLL2025011). Data were collected between January 10, 2026, and February 10, 2026, through Wenjuanxing, a widely used online survey platform in China. The questionnaire was distributed online through university sport teams, sport-related academic programs, and student-athlete networks. The first page of the questionnaire presented detailed information about the study purpose, eligibility criteria, voluntary participation, anonymity, confidentiality, approximate completion time, and the right to withdraw at any time without penalty. All participants were adults. Written informed consent was obtained electronically on the questionnaire homepage, and only participants who confirmed their consent were allowed to proceed to the formal questionnaire.

Several procedures were used to ensure data quality during the survey process. First, all questionnaire items were set as mandatory responses on the Wenjuanxing platform; therefore, missing item-level data were not possible. Second, the questionnaire was designed so that participants could submit only one completed response whenever platform-based restrictions were technically available. Third, participants were instructed to answer independently and honestly according to their actual academic and sport training experiences. Fourth, the questionnaire did not collect personally identifiable information, which helped protect participant privacy and reduce evaluation concerns.

A total of 1,841 questionnaires were initially returned. Before formal statistical analysis, responses were screened according to predefined data quality criteria. Questionnaires were considered invalid and removed if they met one or more of the following conditions: (a) the respondent did not meet the inclusion criteria, such as being younger than 18 years old or not being a collegiate athlete; (b) the completion time was shorter than 180 s, indicating that the participant was unlikely to have read and answered the items carefully; (c) the response pattern indicated careless responding, such as choosing the same response option across all substantive psychological scale items; (d) demographic or sport-related information showed clear logical inconsistency, such as impossible combinations of age, academic year, training experience, and athletic background; or (e) duplicate submissions were identified through platform-based screening information. Because all items were set as required, no questionnaire was excluded due to missing responses. After the exclusion of invalid questionnaires, 1,674 valid responses remained for analysis, yielding an effective response rate of 90.93%.

The final sample consisted of 1,674 Chinese collegiate athletes. Participants’ mean age was 20.61 years (SD = 1.72), with an age range of 18–26 years. Among them, 842 were male athletes (50.30%) and 832 were female athletes (49.70%). Regarding academic year, 846 participants were freshmen (50.54%), 420 were sophomores (25.09%), and 408 were juniors (24.37%). In terms of major type, 1,248 participants were enrolled in sport-related majors (74.55%), whereas 426 were enrolled in non-sport majors (25.45%). Regarding sport type, 832 athletes participated in individual sports (49.70%) and 842 participated in team sports (50.30%). For athletic level, 320 participants were below Level 2 athlete status (19.12%), 810 were Level 2 athletes (48.39%), and 544 were Level 1 athletes or above (32.50%). In terms of years of systematic training, 414 participants had trained for less than 3 years (24.73%), 436 for 3–5 years (26.05%), 401 for 6–8 years (23.95%), and 423 for more than 8 years (25.27%). Regarding highest competition level, 1,106 participants had competed at the provincial level or below (66.07%), whereas 568 had competed above the provincial level (33.93%). Finally, 1,128 participants reported no recent sport-related injury experience (67.38%), while 546 reported recent sport-related injury experience (32.62%). Detailed demographic and sport-related characteristics are presented in Table 1.

**TABLE 1:** Frequency analysis of demographic and sport-related characteristics of participants (*N* = 1,674).

Variable	Category	Frequency (n)	Percentage (%)
Gender	Male	842	50.30
	Female	832	49.70
Academic year	Freshman	846	50.54
	Sophomore	420	25.09
	Junior	408	24.37
Major type	Sport-related major	1,248	74.55
	Non-sport major	426	25.45
Sport type	Individual sport	832	49.70
	Team sport	842	50.30
Athletic level	Below level 2 athlete status	320	19.12
	Level 2 athlete	810	48.39
	Level 1 athlete or Above	544	32.50
Years of systematic training	< 3 years	414	24.73
	3–5 years	436	26.05
	6–8 years	401	23.95
	> 8 years	423	25.27
Highest competition level	Provincial level or below	1,106	66.07
	Above provincial level	568	33.93
Recent sport-related injury experience	No	1,128	67.38
	Yes	546	32.62

“Level 1 athlete or above” includes Level 1 athletes, National Elite Athletes, and International Elite Athletes. “Recent sport-related injury experience” refers to whether participants had experienced a sport-related injury that affected their normal training recently.

### 2.2 Measurements

All measures were administered in Chinese. A five-point Likert-type response format was used for all instruments to maintain consistency across the questionnaire and to reduce response burden among Chinese collegiate athletes. Unless otherwise specified, responses ranged from 1 = “strongly disagree” to 5 = “strongly agree.” For each scale, item scores were averaged after appropriate reverse coding, with higher scores indicating higher levels of the corresponding construct. For instruments originally developed in English, previously validated Chinese versions were used when available. For items without an established Chinese version suitable for the present sport-training context, a translation–back-translation procedure was conducted following standard cross-cultural adaptation recommendations (Brislin, 1970). Specifically, two bilingual researchers independently translated the English items into Chinese, discrepancies were discussed and resolved, and another bilingual researcher back-translated the Chinese version into English. The research team then compared the back-translated version with the original items to ensure semantic equivalence. Before formal data collection, the questionnaire was pilot-tested with a small group of Chinese collegiate athletes to evaluate wording clarity, contextual appropriateness, and completion time. Minor wording adjustments were made when necessary without changing the conceptual meaning of the items.

Future time perspective was measured using the future subscale of the Zimbardo Time Perspective Inventory (ZTPI; Zimbardo and Boyd, 1999). The Future subscale contains 13 items assessing the extent to which individuals tend to plan ahead, consider future consequences, and organize current behavior in relation to future goals. The ZTPI has been examined in Chinese samples, and previous research has provided evidence for the applicability of the Chinese version in the Chinese context (Wang et al., 2015). In the present study, participants responded to the items using a five-point scale. A representative item is: “I believe that a person’s day should be planned ahead each morning.” Higher scores indicated a stronger future time perspective. In the present sample, the Future subscale showed excellent internal consistency and acceptable convergent validity, with Cronbach’s α = 0.942, CR = 0.942, AVE = 0.554, and standardized factor loadings ranging from 0.713 to 0.769.

Self-control was assessed using the 13-item Brief Self-Control Scale developed by Tangney et al. (2004). The scale measures general trait self-control, including the ability to resist temptation, regulate impulses, and act in accordance with long-term goals. The scale has been used as a unidimensional measure in Chinese samples; for example, Li et al. (2017) used the 13-item version in their study on adolescents and reported acceptable internal consistency. In the present study, all items were rated on a five-point scale. A representative item is: “I am good at resisting temptation.” Following the Chinese application by Li et al. (2017), items 1, 6, 9, and 11 were reverse-coded where appropriate. To make the interpretation consistent with the theoretical model, final scores were oriented so that higher scores represented higher levels of self-control. In the present sample, the Brief Self-Control Scale showed excellent internal consistency and acceptable convergent validity, with Cronbach’s α = 0.948, CR = 0.949, AVE = 0.588, and standardized factor loadings ranging from 0.679 to 0.856.

Achievement goal orientations were assessed using two subscales from the Achievement Goal Questionnaire for Sport (AGQ-S; Conroy et al., 2003): mastery-approach goal orientation and performance-avoidance goal orientation. The original AGQ-S was developed based on the 2 × 2 achievement goal framework and includes four dimensions: mastery-approach, mastery-avoidance, performance-approach, and performance-avoidance goal orientations. In the present study, only the mastery-approach and performance-avoidance subscales were used because they represent two theoretically contrasting motivational orientations that are particularly relevant to the proposed model. Mastery-approach goal orientation reflects an adaptive tendency to strive for personal improvement, skill mastery, and optimal performance, whereas performance-avoidance goal orientation reflects a concern with avoiding poor performance or doing worse than others. The two selected subscales contained six items in total, with three items for mastery-approach goal orientation and three items for performance-avoidance goal orientation. Participants responded to all items on a five-point Likert scale ranging from 1 = “strongly disagree” to 5 = “strongly agree.” A representative item for mastery-approach goal orientation is: “It is important to me to perform as well as I possibly can.” A representative item for performance-avoidance goal orientation is: “I just want to avoid performing worse than others.” Higher scores on each subscale indicated a stronger tendency toward the corresponding achievement goal orientation. Because the original AGQ-S was developed in English and no single simplified Chinese version was adopted as the standard measure for Chinese collegiate athletes in the present training context, a translation–back-translation procedure was conducted before data collection. Two bilingual researchers independently translated the selected items into Chinese. Discrepancies were discussed and resolved by the research team. Another bilingual researcher then back-translated the Chinese version into English, and the back-translated items were compared with the original English items to ensure semantic equivalence. The translated items were further reviewed by experts in sport psychology and physical education to evaluate their clarity and contextual appropriateness for Chinese collegiate athletes. Before the formal survey, the items were pilot-tested with a small group of collegiate athletes, and minor wording adjustments were made when necessary without changing the original meaning of the items. The two subscales were scored separately rather than combined into an overall achievement goal orientation score, because mastery-approach and performance-avoidance goal orientations represent qualitatively different motivational tendencies. In the formal analysis, the two-factor structure of the selected AGQ-S subscales was examined using confirmatory factor analysis before testing the hypothesized model. In the present sample, the mastery-approach subscale showed good internal consistency and acceptable convergent validity, with Cronbach’s α = 0.842, CR = 0.844, AVE = 0.644, and standardized factor loadings ranging from 0.772 to 0.860. The performance-avoidance subscale showed acceptable internal consistency and convergent validity, with Cronbach’s α = 0.794, CR = 0.796, AVE = 0.565, and standardized factor loadings ranging from 0.721 to 0.779.

Training procrastination was measured using a training-context-adapted version of the Irrational Procrastination Scale (IPS; Steel, 2002). The IPS is a 9-item measure of irrational procrastination and has been validated in Chinese college student samples, with evidence supporting its one-factor structure and measurement invariance across gender (Shaw and Zhang, 2021). Because the original IPS assesses general procrastination, the present study adapted the wording to the sport training context. Specifically, participants were instructed to respond according to their daily sport training experiences, and general task-related expressions were modified into training-related expressions where necessary. For example, the original general item “I delay tasks beyond what is reasonable” was adapted as “I delay training tasks beyond what is reasonable.” This adaptation was intended to preserve the original meaning of irrational delay while making the item directly relevant to sport training. The adapted items were reviewed by bilingual researchers and sport psychology experts to ensure semantic equivalence and contextual appropriateness. Participants responded on a five-point scale. Reverse-coded items were recoded according to the IPS scoring procedure, and higher scores indicated higher levels of training procrastination. In the present sample, the training-context-adapted IPS showed excellent internal consistency and acceptable convergent validity, with Cronbach’s α = 0.924, CR = 0.925, AVE = 0.580, and standardized factor loadings ranging from 0.725 to 0.806.

### 2.3 Data analysis

Data were analyzed using IBM SPSS Statistics 29.0, AMOS 26.0, and the PROCESS macro for SPSS. Before conducting the main analyses, the dataset was screened for accuracy, invalid responses, and distributional properties. Because all questionnaire items were set as mandatory on the online survey platform, there were no missing item-level data. Descriptive statistics, including means and standard deviations, were calculated for the main study variables. Skewness and kurtosis were examined to evaluate distributional normality. Absolute skewness values below 2.00 and absolute kurtosis values below 7.00 were considered acceptable for subsequent analyses (Curran et al., 1996).

Demographic and sport-related variables were handled differently across analyses according to the purpose of each statistical procedure. In the regression-based analyses, gender, academic year, major type, sport type, athletic level, years of systematic training, highest competition level, and recent sport-related injury experience were included as control variables to examine whether the focal associations remained evident after accounting for key background characteristics. In contrast, the structural equation mediation model did not include these demographic and sport-related covariates. This decision was made because the SEM was intended to test the core latent-variable mediation pathway among future time perspective, self-control, and training procrastination while accounting for measurement error in the focal psychological constructs. Thus, the SEM provided a parsimonious test of the theoretically specified mediation process, whereas the regression-based models provided covariate-adjusted evidence for the direct and moderated associations.

First, common method bias was examined because all variables were measured using self-report questionnaires. Harman’s single-factor test was conducted using unrotated principal component analysis. Common method bias was considered unlikely to be a serious concern if the first unrotated factor accounted for less than 40% of the total variance. In addition, a single-factor confirmatory factor model was compared with a series of competing measurement models. If the single-factor model showed poor fit and the hypothesized multi-factor model demonstrated substantially better fit, common method bias was considered unlikely to seriously threaten the validity of the findings (Podsakoff et al., 2003).

Second, reliability and convergent validity were assessed for all study variables. Internal consistency was evaluated using Cronbach’s alpha coefficients. Values of 0.70 or higher were considered acceptable, values of 0.80 or higher were considered good, and values of 0.90 or higher were considered excellent. Confirmatory factor analysis was conducted using AMOS 26.0 to evaluate the measurement structure of the main constructs, including future time perspective, self-control, mastery-approach goal orientation, performance-avoidance goal orientation, and training procrastination. Model fit was evaluated using the chi-square/degrees of freedom ratio, comparative fit index, Tucker–Lewis index, standardized root mean square residual, and root mean square error of approximation. The following criteria were used to evaluate acceptable model fit: chi-square/degrees of freedom ratio < 5.00, CFI and TLI ≥ 0.90, and SRMR and RMSEA ≤ 0.08. More stringent criteria, including CFI and TLI ≥ 0.95 and SRMR and RMSEA ≤ 0.06, were interpreted as indicating good model fit (Hu and Bentler, 1999). Standardized factor loadings of 0.50 or higher were considered acceptable, whereas loadings of 0.70 or higher were considered desirable. Composite reliability values of 0.70 or higher and average variance extracted values of 0.50 or higher were used as evidence of convergent validity (Fornell and Larcker, 1981; Hair et al., 2019).

Third, Pearson correlation analysis was conducted to examine the bivariate associations among future time perspective, self-control, mastery-approach goal orientation, performance-avoidance goal orientation, and training procrastination. The magnitude of correlations was interpreted using conventional criteria: correlations around 0.10 were considered small, around 0.30 moderate, and 0.50 or above large. Discriminant validity was evaluated using both the Fornell–Larcker criterion and the heterotrait–monotrait ratio of correlations. For the Fornell–Larcker criterion, discriminant validity was considered acceptable when the square root of the AVE for each construct was greater than its correlations with other constructs (Fornell and Larcker, 1981). For HTMT, values below 0.85 were interpreted as indicating satisfactory discriminant validity, whereas values below 0.90 were considered acceptable for conceptually related constructs (Henseler et al., 2015; Hair et al., 2019).

Fourth, hierarchical multiple regression analysis was performed to examine the predictive effects of future time perspective and self-control on training procrastination after controlling for demographic and sport-related variables. Training procrastination was entered as the dependent variable. In the first step, control variables were entered, including gender, academic year, major type, sport type, athletic level, years of systematic training, highest competition level, and recent sport-related injury experience. In the second step, future time perspective was added to examine its association with training procrastination after accounting for these background variables. In the third step, self-control was further added to examine whether future time perspective remained significant after accounting for the proposed mediator. Multicollinearity was assessed using variance inflation factors, with VIF values below 5.00 indicating that multicollinearity was not a serious concern. Binary control variables were dummy-coded, and ordered categorical variables were entered according to their ordinal coding.

Fifth, the mediating role of self-control was tested using structural equation modeling in AMOS 26.0. Future time perspective was specified as the independent variable, self-control as the mediator, and training procrastination as the dependent variable. The SEM mediation model did not include demographic or sport-related covariates because its purpose was to estimate the core latent-variable mediation pathway among the focal psychological constructs. Model fit was evaluated using the chi-square/degrees of freedom ratio, CFI, TLI, SRMR, and RMSEA. The significance of the indirect effect was further examined using a bootstrap procedure with 5,000 resamples. The mediating effect was considered statistically significant when the 95% bootstrap confidence interval did not include zero.

Sixth, the moderating effects of achievement goal orientations were tested using Hayes’ PROCESS macro Model 5 (Hayes, 2022). In PROCESS Model 5, self-control was specified as the mediator, and the moderator was specified on the direct path from future time perspective to training procrastination. Because PROCESS Model 5 allows one moderator at a time, two separate models were tested. In the first model, future time perspective was entered as the independent variable, self-control as the mediator, training procrastination as the dependent variable, and mastery-approach goal orientation as the moderator. In the second model, performance-avoidance goal orientation was entered as the moderator using the same model specification. Gender, academic year, major type, sport type, athletic level, years of systematic training, highest competition level, and recent sport-related injury experience were included as control variables in both models. Continuous predictors involved in interaction terms were mean-centered to reduce nonessential multicollinearity and facilitate interpretation. Moderation effects were evaluated by examining the significance and direction of the interaction term between future time perspective and the moderator. Conditional effects were estimated at one standard deviation below the mean, the mean, and one standard deviation above the mean of the moderator. Simple slope plots were created to illustrate the interaction patterns (Wen and Ye, 2014).

Finally, to address the potential conceptual and empirical overlap between mastery-approach and performance-avoidance goal orientations, an additional simultaneous-interaction regression model was conducted as a robustness analysis. In this model, training procrastination was specified as the dependent variable. Future time perspective, self-control, mastery-approach goal orientation, performance-avoidance goal orientation, the interaction between future time perspective and mastery-approach goal orientation, the interaction between future time perspective and performance-avoidance goal orientation, and all demographic and sport-related control variables were entered simultaneously. Future time perspective, self-control, mastery-approach goal orientation, and performance-avoidance goal orientation were mean-centered before the product terms were computed. This robustness analysis was used to examine whether each moderation pattern remained evident after accounting for the other achievement goal orientation. Conditional effects of future time perspective on training procrastination were estimated at one standard deviation below the mean, the mean, and one standard deviation above the mean of each moderator. When probing mastery-approach goal orientation, performance-avoidance goal orientation was held at its mean. When probing performance-avoidance goal orientation, mastery-approach goal orientation was held at its mean. Self-control and all control variables were held at their sample means.

The level of statistical significance was set at *p* < 0.05 for all analyses. Standardized coefficients were reported for correlation, hierarchical regression, and structural equation modeling analyses where appropriate, whereas unstandardized coefficients were reported for PROCESS moderation analyses and the simultaneous-interaction regression model. Standard errors, confidence intervals, and effect estimates were reported where applicable.

## 3 Results

### 3.1 Common method bias test

Because all variables were measured using self-report questionnaires, common method bias was examined before testing the hypothesized model. Harman’s single-factor test showed that five factors had eigenvalues greater than 1, accounting for 62.93% of the total variance. The first factor explained 27.94% of the variance, which was below the commonly used threshold of 40%. This result suggested that no single factor accounted for the majority of covariance among the measured variables.

Confirmatory factor analyses were also conducted to compare the fit of the single-factor model with several alternative measurement models. As shown in Table 2, the one-factor model showed poor fit to the data, χ^2^/df = 32.755, CFI = 0.413, TLI = 0.382, SRMR = 0.188, RMSEA = 0.138, 90% CI [0.123, 0.144]. In contrast, the hypothesized five-factor model demonstrated good model fit, χ^2^/df = 2.745, CFI = 0.968, TLI = 0.966, SRMR = 0.021, RMSEA = 0.032, 90% CI [0.031, 0.034]. Although the four-factor model also reached an acceptable level of fit, the five-factor model showed better fit across all indices. These findings indicated that the main constructs were empirically distinguishable and argued against a dominant single-factor explanation of the covariance structure; however, other forms of common-method variance associated with the concurrent self-report design cannot be ruled out.

**TABLE 2:** Comparison of competing measurement models for common method bias and construct distinctiveness.

Model	χ^2^/df	CFI	TLI	SRMR	RMSEA (90% CI)
One-factor	32.755	0.413	0.382	0.188	0.138 (0.123–0.144)
Two-factor	25.682	0.544	0.519	0.174	0.121 (0.109–0.127)
Three-factor	21.435	0.624	0.602	0.163	0.111 (0.099–0.116)
Four-factor	3.724	0.950	0.947	0.027	0.040 (0.036–0.042)
Five-factor	2.745	0.968	0.966	0.021	0.032 (0.031–0.034)

FTP, future time perspective; SC, self-control; MAp, mastery-approach goal orientation; PAv, performance-avoidance goal orientation; TPro, training procrastination. In the one-factor model, all items were loaded onto a single common factor. In the two-factor model, FTP, SC, MAp, and PAv items were loaded onto one factor, and TPro items were loaded onto another factor. In the three-factor model, FTP and SC items were loaded onto one self-regulatory factor, MAp and PAv items were loaded onto one achievement goal orientation factor, and TPro items were loaded onto a separate factor. In the four-factor model, FTP, SC, and TPro were specified as separate factors, whereas MAp and PAv were combined into one achievement goal orientation factor. In the five-factor model, FTP, SC, TPro, MAp, and PAv were specified as five separate but correlated latent factors. CFI, comparative fit index; TLI, Tucker–Lewis index; SRMR, standardized root mean square residual; RMSEA, root mean square error of approximation; CI, confidence interval.

### 3.2 Descriptive statistics, reliability, and convergent validity

Descriptive statistics, internal consistency, and convergent validity indices for the study variables are presented in Table 3. Cronbach’s alpha coefficients ranged from 0.794 to 0.948, exceeding the recommended threshold of 0.70. Standardized factor loadings ranged from 0.679 to 0.860. Although the lowest factor loading for self-control was slightly below 0.70, all loadings exceeded the acceptable threshold of 0.50. Composite reliability values ranged from 0.796 to 0.949, and average variance extracted values ranged from 0.554 to 0.644. These results indicated that the measurement scales had satisfactory internal consistency and convergent validity.

**TABLE 3:** Descriptive statistics, reliability, and convergent validity of the study variables.

Variable	Mean	SD	α	Std. factor loadings	CR	AVE
Future time perspective	3.597	0.883	0.942	0.713–0.769	0.942	0.554
Self-control	3.435	0.939	0.948	0.679–0.856	0.949	0.588
Mastery-approach goal orientation	3.494	1.025	0.842	0.772–0.860	0.844	0.644
Performance-avoidance goal orientation	2.536	1.015	0.794	0.721–0.779	0.796	0.565
Training procrastination	2.403	0.917	0.924	0.725–0.806	0.925	0.580

SD, standard deviation; α, Cronbach’s alpha; CR, composite reliability; AVE, average variance extracted. Standardized factor loadings are reported as the range of item loadings for each latent construct. Higher scores indicate higher levels of the corresponding construct.

A separate one-factor CFA was conducted for the training-context-adapted IPS. The model showed good fit, χ^2^/df = 2.810, CFI = 0.994, TLI = 0.993, SRMR = 0.013, and RMSEA = 0.033, 90% CI [0.024, 0.042]. Standardized factor loadings ranged from 0.725 to 0.806. Together with Cronbach’s α = 0.924, CR = 0.925, and AVE = 0.580, these findings provided preliminary support for the internal consistency and internal structure of the adapted measure in the present sample.

### 3.3 Correlation analysis and discriminant validity

Pearson correlation analysis was conducted to examine the bivariate associations among the study variables. As shown in Table 4, future time perspective was positively correlated with self-control, *r* = 0.202, *p* < 0.001, and negatively correlated with training procrastination, *r* = −0.378, *p* < 0.001. Self-control was also negatively correlated with training procrastination, *r* = −0.344, *p* < 0.001.

**TABLE 4:** Correlations among study variables and square roots of AVE.

Variable	FTP	SC	MAp	PAv	TPro
FTP	0.744	0.767	0.802	0.752	0.762
SC	0.202***				
MAp	0.007	0.113***			
PAv	0.024	−0.097***	−0.507***		
TPro	−0.378***	−0.344***	−0.051*	0.033	

FTP, future time perspective; SC, self-control; MAp, mastery-approach goal orientation; PAv, performance-avoidance goal orientation; TPro, training procrastination. AVE, average variance extracted. Diagonal elements in bold represent the square roots of AVE.

*p < 0.05, ***p < 0.001.

Regarding achievement goal orientations, mastery-approach goal orientation was positively correlated with self-control, *r* = 0.113, *p* < 0.001, and weakly but significantly negatively correlated with training procrastination, *r* = −0.051, *p* < 0.05. Performance-avoidance goal orientation was negatively correlated with self-control, *r* = −0.097, *p* < 0.001, but was not significantly correlated with training procrastination, *r* = 0.033, *p* > 0.05. Mastery-approach and performance-avoidance goal orientations were moderately and negatively correlated, *r* = −0.507, *p* < 0.001, indicating that the two motivational orientations were related but empirically distinguishable.

Discriminant validity was first evaluated using the Fornell–Larcker criterion. As shown in Table 4, the square roots of AVE ranged from 0.744 to 0.802 and were greater than the corresponding inter-construct correlations for each variable. These results indicated acceptable discriminant validity among the five constructs.

Discriminant validity was further examined using the heterotrait–monotrait ratio of correlations. As shown in Table 5, all HTMT values ranged from 0.025 to 0.620, which were below the recommended threshold of 0.85. This provided further evidence that the constructs had satisfactory discriminant validity.

**TABLE 5:** Heterotrait–monotrait ratio of correlations (HTMT) among study variables.

Variable	FTP	SC	MAp	PAv	TPro
FTP	–	–	–	–	–
SC	0.213				
MAp	0.025	0.126			
PAv	0.038	0.111	0.620		
TPro	0.405	0.367	0.059	0.046	

FTP, future time perspective; SC, self-control; MAp, mastery-approach goal orientation; PAv, performance-avoidance goal orientation; TPro, training procrastination. HTMT, heterotrait–monotrait ratio of correlations. Values below 0.85 indicate satisfactory discriminant validity.

### 3.4 Hierarchical multiple regression analysis

Hierarchical multiple regression analysis was conducted to examine the associations of future time perspective and self-control with training procrastination after controlling for demographic and sport-related variables. As shown in Table 6, the control-only model was significant, *R*^2^ = 0.092, adjusted *R*^2^ = 0.088, *F* = 21.166, *p* < 0.001, indicating that demographic and sport-related variables explained 9.2% of the variance in training procrastination.

**TABLE 6:** Hierarchical multiple regression analysis predicting training procrastination.

Variables	Model A (controls only)		Model B (+Future time perspective)		Model C +Self-control)	
	β	*t*	β	*t*	β	*t*
Control variables						
Gender	−0.075**	−3.212	−0.042	−1.896	−0.049*	−2.253
Academic year	−0.141***	−5.939	−0.113***	−5.021	−0.080***	−3.656
Major type	0.012	0.517	0.005	0.211	0.009	0.425
Sport type	0.019	0.791	0.017	0.764	0.014	0.642
Athletic level	−0.169***	−7.176	−0.139***	−6.241	−0.109***	−5.005
Years of systematic training	−0.138***	−5.838	−0.087***	−3.831	−0.061**	−2.782
Highest competition level	0.012	0.513	0.012	0.546	0.016	0.755
Recent sport-related injury experience	0.025	1.089	0.033	1.496	0.029	1.376
Independent variable						
Future time perspective			−0.328***	−14.398	−0.290***	−13.029
Mediator						
Self-control					−0.242***	−10.824
Model summary						
*R* ^2^	0.092		0.193		0.246	
Adjusted R^2^	0.088		0.189		0.241	
*F*	21.166***		44.180***		54.253***	

Standardized coefficients (β) are reported. The dependent variable was training procrastination. Model A included only control variables. Model B added future time perspective. Model C further added self-control.

*p < 0.05, **p < 0.01, ***p < 0.001.

After future time perspective was entered in Model B, the explained variance increased to 19.3%, *R*^2^ = 0.193, adjusted *R*^2^ = 0.189, *F* = 44.180, *p* < 0.001. Future time perspective was significantly and negatively associated with training procrastination, β = −0.328, *t* = −14.398, *p* < 0.001. This result supported H1.

In Model C, self-control was added to the regression model. The model explained 24.6% of the variance in training procrastination, *R*^2^ = 0.246, adjusted *R*^2^ = 0.241, *F* = 54.253, *p* < 0.001. Self-control was negatively associated with training procrastination, β = −0.242, *t* = −10.824, *p* < 0.001. Future time perspective remained significantly and negatively associated with training procrastination after self-control was included, although its standardized coefficient decreased from β = −0.328 to β = −0.290. This pattern provided preliminary evidence for the partial mediating role of self-control.

### 3.5 Mediation analysis

A structural equation model was constructed to examine whether self-control mediated the association between future time perspective and training procrastination. The model demonstrated good fit to the data, χ^2^/df = 3.385, CFI = 0.965, TLI = 0.963, SRMR = 0.021, RMSEA = 0.038, 90% CI [0.036, 0.040].

**FIGURE 2:**
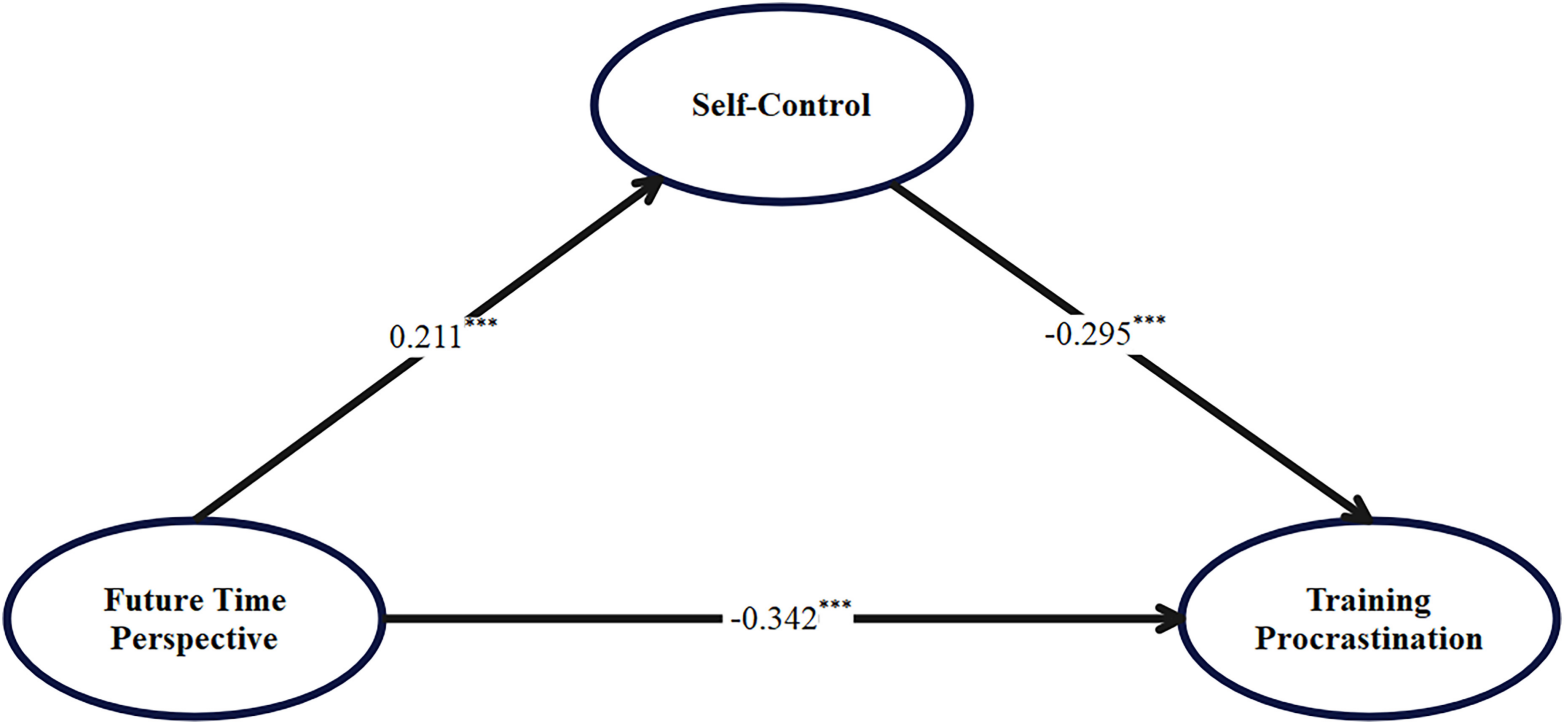
Standardized path coefficients of the structural mediation model. Standardized path coefficients are reported. The figure presents the simplified structural mediation pathway from future time perspective to training procrastination through self-control. For readability, item-level indicators, measurement errors, and standardized factor loadings are not shown in the main figure. The full latent-variable measurement and structural model is presented in Supplementary Figure 1. ***p < 0.001.

As shown in Figure 2, future time perspective was positively associated with self-control, β = 0.211, *p* < 0.001. Future time perspective was also negatively associated with training procrastination, β = −0.342, *p* < 0.001. In addition, self-control was negatively associated with training procrastination, β = −0.295, *p* < 0.001. The model explained 4.5% of the variance in self-control and 24.7% of the variance in training procrastination. To improve readability, Figure 2 presents only the key structural paths among the latent variables; the full measurement model with item-level indicators and standardized factor loadings is provided in Supplementary Figure 1.

A bootstrap analysis with 5,000 resamples was conducted to test the significance of the indirect effect. As shown in Table 7, the total effect of future time perspective on training procrastination was significant, β = −0.405, Boot SE = .023, *p* < 0.001, 95% CI [−0.449, −0.358]. The direct effect remained significant after self-control was included, β = −0.342, Boot SE = 0.024, *p* < 0.001, 95% CI [−0.387, −0.294]. The indirect effect through self-control was also significant, β = −0.063, Boot SE = 0.009, *p* < 0.001, 95% CI [−0.080, −0.046]. Because the 95% bootstrap confidence interval for the indirect effect did not include zero, self-control partially mediated the relationship between future time perspective and training procrastination. The indirect effect accounted for 15.56% of the total effect. These findings supported H2.

**TABLE 7:** Total, direct and indirect effects in the mediation model.

Path	β	Boot SE	*p*	Boot LLCI	Boot ULCI	Ratio (%)
Direct effect						
Future time perspective→Training procrastination	−0.342***	0.024	< .001	−0.387	−0.294	84.44
Indirect effects						
Future time perspective→Self control→Training procrastination	−0.063***	0.009	< .001	−0.080	−0.046	15.56
Total effect						
Future time perspective→Training procrastination	−0.405***	0.023	< .001	−0.449	−0.358	100

Standardized coefficients are reported. Boot SE, bootstrap standard error; Boot LLCI, lower limit of the 95% bootstrap confidence interval; Boot ULCI, upper limit of the 95% bootstrap confidence interval. Bootstrap estimates were based on 5,000 resamples using the bias-corrected percentile bootstrap method.

***p < 0.001.

### 3.6 Moderation and robustness analyses

Planned moderation analyses were conducted using PROCESS Model 5 to examine whether mastery-approach goal orientation and performance-avoidance goal orientation moderated the direct association between future time perspective and training procrastination. As shown in Table 8, Model D tested mastery-approach goal orientation as the moderator, and Model E tested performance-avoidance goal orientation as the moderator.

**TABLE 8:** Planned PROCESS Model 5 analyses testing the separate moderating roles of mastery-approach and performance-avoidance goal orientations.

Variables	Model D			Model E		
	*B*	SE	*t*	*B*	SE	*t*
Control variables						
Gender	−0.084*	0.039	−2.121	−0.086*	0.039	−2.171
Academic year	−0.091***	0.024	−3.720	−0.089***	0.024	−3.675
Major type	0.019	0.045	0.433	0.018	0.045	0.407
Sport type	0.026	0.039	0.669	0.027	0.039	0.680
Athletic level	−0.139***	0.028	−4.880	−0.139***	0.028	−4.917
Years of systematic training	−0.051**	0.018	−2.845	−0.049**	0.018	−2.712
Highest competition level	0.035	0.041	0.836	0.031	0.041	0.747
Recent sport-related injury experience	0.067	0.042	1.596	0.063	0.042	1.516
Independent variable						
Future time perspective	−0.303***	0.023	−13.113	−0.304***	0.023	−13.122
Mediator						
Self-control	−0.242***	0.022	−11.053	−0.239***	0.022	−10.927
Moderator						
Mastery-approach goal orientation	0.005	0.019	0.259			
Performance-avoidance goal orientation				0.001	0.019	0.030
Interaction term						
Future time perspective × Mastery-approach goal orientation	−0.072***	0.021	−3.406			
Future time perspective × Performance-avoidance goal orientation				0.058**	0.021	2.719
Model summary						
R^2^	0.251			0.249		
Adjust R^2^	0.245			0.243		
*F*	46.451***			45.974***		

Unstandardized regression coefficients are reported. The dependent variable was training procrastination. Model D tested mastery-approach goal orientation as the moderator. Model E tested performance-avoidance goal orientation as the moderator. Gender, academic year, major type, sport type, athletic level, years of systematic training, highest competition level, and recent sport-related injury experience were included as control variables. *SE*, standard error.

*p < 0.05, **p < 0.01, ***p < 0.001.

In Model D, the interaction between future time perspective and mastery-approach goal orientation was significant, *B* = −0.072, SE = 0.021, *t* = −3.406, *p* < 0.001. This result indicated that the negative association between future time perspective and training procrastination was stronger among athletes with higher mastery-approach goal orientation. Thus, the planned PROCESS analysis supported H3.

In Model E, the interaction between future time perspective and performance-avoidance goal orientation was also significant, *B* = 0.058, SE = 0.021, *t* = 2.719, *p* < 0.01. The positive interaction coefficient indicated that performance-avoidance goal orientation weakened the negative association between future time perspective and training procrastination. Thus, the planned PROCESS analysis provided initial support for H4.

To address the potential overlap between mastery-approach and performance-avoidance goal orientations, an additional simultaneous-interaction regression model was conducted. In this model, mastery-approach goal orientation, performance-avoidance goal orientation, and both interaction terms were entered simultaneously, together with future time perspective, self-control, and all control variables. As shown in Table 9, the interaction between future time perspective and mastery-approach goal orientation remained significant, *B* = −0.058, SE = 0.025, *t* = −2.338, *p* = 0.020, 95% CI [−0.107, −0.009]. In contrast, the interaction between future time perspective and performance-avoidance goal orientation was not significant, *B* = 0.028, SE = 0.025, *t* = 1.098, *p* = 0.272, 95% CI [−0.022, 0.077]. These results indicated that the moderating role of mastery-approach goal orientation was robust after accounting for performance-avoidance goal orientation, whereas the unique moderating role of performance-avoidance goal orientation was not robust.

**TABLE 9:** Simultaneous-interaction regression model predicting training procrastination.

Predictor	*B*	SE	*t*	*p*	95% CI lower	95% CI upper	VIF
Future time perspective	−0.304***	0.023	−13.136	< 0.001	−0.349	−0.259	1.099
Self-control	−0.242***	0.022	−11.042	< 0.001	−0.285	−0.199	1.118
Mastery-approach goal orientation	0.007	0.022	0.308	0.758	−0.037	0.051	1.381
Performance-avoidance goal orientation	0.005	0.022	0.216	0.829	−0.039	0.049	1.358
Future time perspective × Mastery-approach goal orientation	−0.058*	0.025	−2.338	0.02	−0.107	−0.009	1.393
Future time perspective × Performance-avoidance goal orientation	0.028	0.025	1.098	0.272	−0.022	0.077	1.386

*N* = 1,674. Unstandardized coefficients are reported. The dependent variable was training procrastination. Future time perspective, self-control, mastery-approach goal orientation, performance-avoidance goal orientation, and both interaction terms were entered simultaneously. Gender, academic year, major type, sport type, athletic level, years of systematic training, highest competition level, and recent sport-related injury experience were included as control variables but are not displayed for parsimony. FTP, SC, MAp, and PAv were mean-centered before product terms were computed. CI, confidence interval; VIF, variance inflation factor.

*p < 0.05, ***p < 0.001.

**FIGURE 3:**
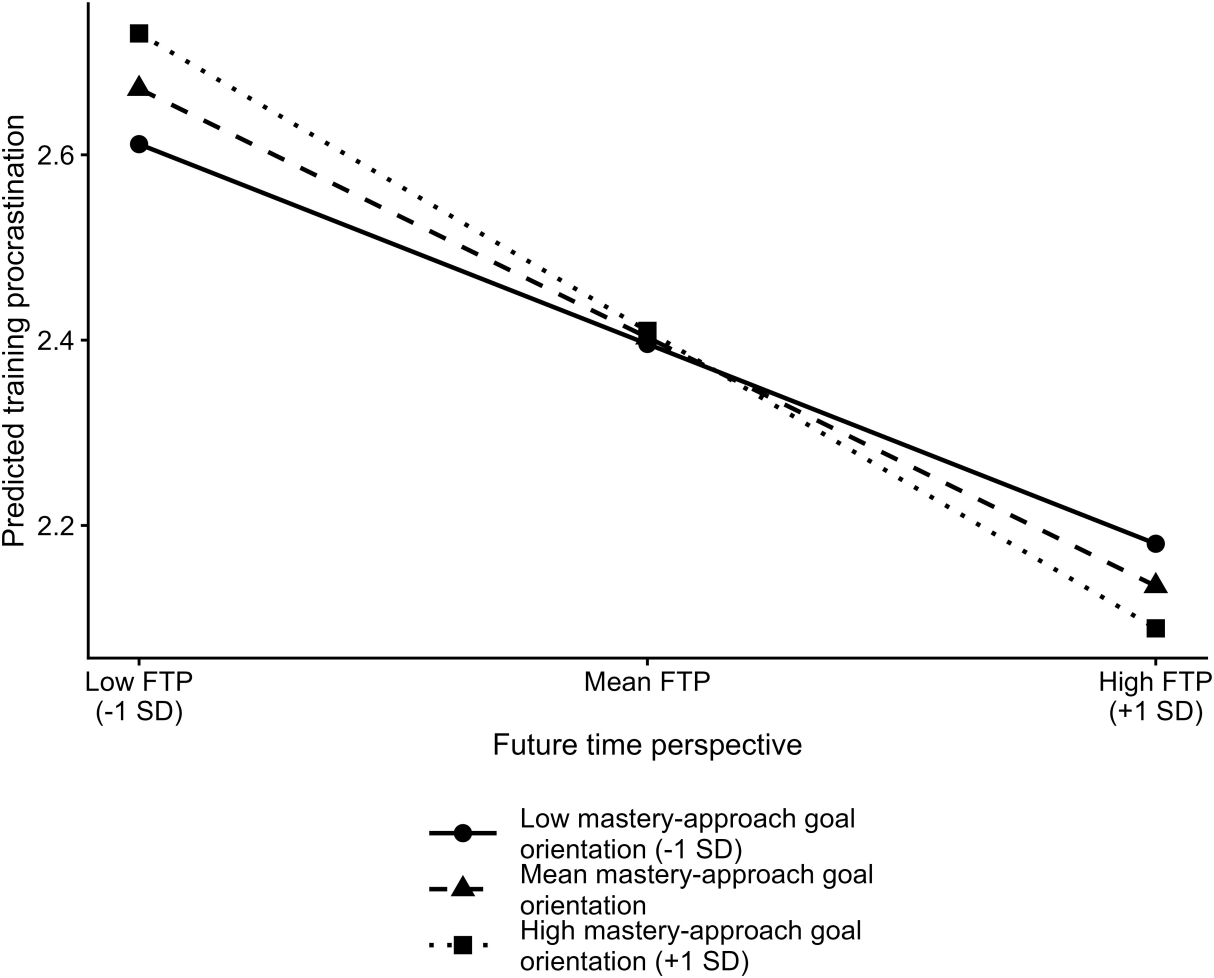
Conditional association between future time perspective and predicted training procrastination at low, mean, and high levels of mastery-approach goal orientation. Predicted values were estimated from the simultaneous-interaction regression model. Low and high levels of mastery-approach goal orientation represent one standard deviation below and above the mean, respectively. Performance-avoidance goal orientation, self-control, and all control variables were held at their sample means.

Conditional effects based on the simultaneous-interaction model are presented in Table 10. When performance-avoidance goal orientation was held at its mean, the negative association between future time perspective and training procrastination became stronger as mastery-approach goal orientation increased. Specifically, the conditional effect of future time perspective on training procrastination was *B* = −0.244 at low mastery-approach goal orientation, *B* = −0.304 at mean mastery-approach goal orientation, and *B* = −0.364 at high mastery-approach goal orientation, all *p*s < 0.001. This pattern is illustrated in Figure 3.

**TABLE 10:** Conditional effects of future time perspective on training procrastination at low, mean, and high levels of goal orientations.

Moderator	Level of moderator	Moderator value	Effect of FTP on TPro	SE	*t*	*p*	95% CI lower	95% CI upper
MAp	−1 SD	2.469	−0.244***	0.034	−7.100	< 0.001	−0.312	−0.177
MAp	Mean	3.494	−0.304***	0.023	−13.136	< 0.001	−0.349	−0.259
MAp	+1 SD	4.519	−0.364***	0.035	−10.536	< 0.001	−0.431	−0.296
PAv	−1 SD	1.521	−0.332***	0.035	−9.506	< 0.001	−0.400	−0.263
PAv	Mean	2.536	−0.304***	0.023	−13.136	< 0.001	−0.349	−0.259
PAv	+1 SD	3.551	−0.276***	0.034	−8.151	< 0.001	−0.342	−0.210

Conditional effects were derived from the simultaneous-interaction OLS regression model. When probing MAp, PAv was held at its mean; when probing PAv, MAp was held at its mean. SC and control variables were held at their sample means. Gender, academic year, major type, sport type, athletic level, years of systematic training, highest competition level, and recent sport-related injury experience were included as control variables in the simultaneous-interaction model. FTP, future time perspective; MAp, mastery-approach goal orientation; PAv, performance-avoidance goal orientation; TPro, training procrastination; CI, confidence interval.

***p < 0.001.

**FIGURE 4:**
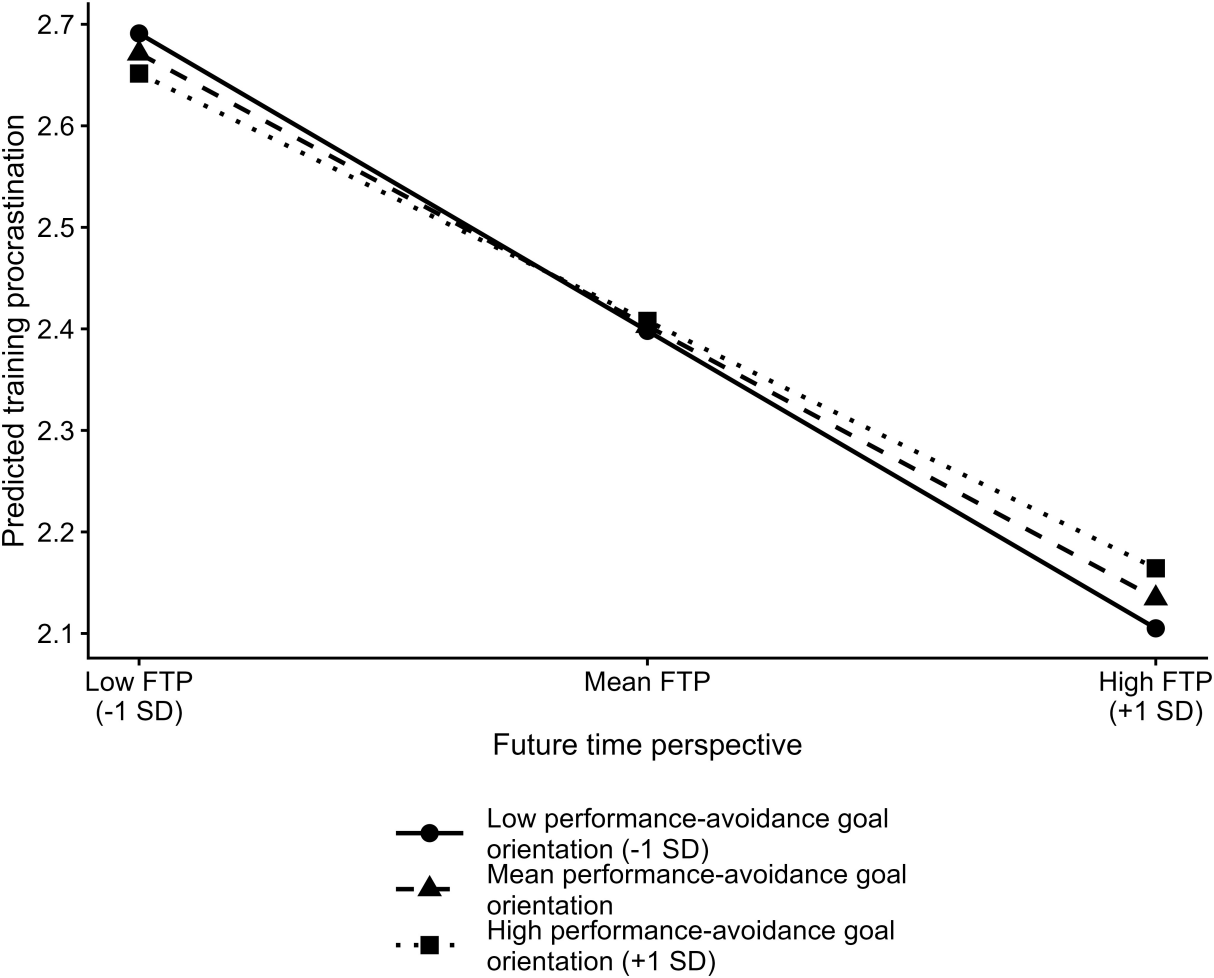
Conditional association between future time perspective and predicted training procrastination at low, mean, and high levels of performance-avoidance goal orientation. Predicted values were estimated from the simultaneous-interaction regression model. Low and high levels of performance-avoidance goal orientation represent one standard deviation below and above the mean, respectively. Mastery-approach goal orientation, self-control, and all control variables were held at their sample means. The plotted slopes are presented to illustrate the conditional pattern; the statistical interpretation should be based on the corresponding interaction term in the simultaneous-interaction model.

When mastery-approach goal orientation was held at its mean, future time perspective remained negatively associated with training procrastination across all levels of performance-avoidance goal orientation. The conditional effect was *B* = −0.332 at low performance-avoidance goal orientation, *B* = −0.304 at mean performance-avoidance goal orientation, and *B* = −0.276 at high performance-avoidance goal orientation, all *p*s < 0.001. Although the plotted pattern in Figure 4 showed a descriptively weaker negative association at higher levels of performance-avoidance goal orientation, the corresponding interaction term was not significant in the simultaneous-interaction model. Therefore, this pattern should be interpreted as a descriptive tendency rather than robust evidence of an independent moderation effect.

Taken together, the planned PROCESS analyses indicated that mastery-approach goal orientation strengthened, whereas performance-avoidance goal orientation weakened, the negative association between future time perspective and training procrastination. However, the simultaneous-interaction robustness model qualified this pattern. The mastery-approach interaction remained significant after accounting for performance-avoidance goal orientation, whereas the performance-avoidance interaction became non-significant after accounting for mastery-approach goal orientation. Therefore, H3 was robustly supported, whereas H4 received only limited support and should be interpreted cautiously.

## 4 Discussion

### 4.1 Summary of main findings

This study examined the relationships among future time perspective, self-control, mastery-approach and performance-avoidance goal orientations, and training procrastination among Chinese collegiate athletes within the context of sustainable athlete development. Several findings emerged. First, future time perspective was negatively associated with training procrastination. Chinese collegiate athletes who reported stronger future-oriented thinking tended to report lower levels of irrational delay in training. Second, self-control partially mediated the association between future time perspective and training procrastination. Specifically, future time perspective was positively associated with self-control, and self-control was negatively associated with training procrastination. Third, the planned PROCESS analyses showed that mastery-approach goal orientation strengthened the negative association between future time perspective and training procrastination, whereas performance-avoidance goal orientation weakened this association in the separate moderation model.

The additional simultaneous-interaction model further examined whether these two moderation effects remained evident after accounting for the overlap between mastery-approach and performance-avoidance goal orientations. This robustness analysis showed that the interaction between future time perspective and mastery-approach goal orientation remained significant, whereas the interaction between future time perspective and performance-avoidance goal orientation was attenuated and became non-significant. Therefore, the findings provide robust support for the moderating role of mastery-approach goal orientation, while the moderating role of performance-avoidance goal orientation should be interpreted more cautiously.

Taken together, these findings suggest that training procrastination among collegiate athletes should not be understood merely as a problem of poor time management. Rather, it appears to be associated with how athletes think about the future, how they regulate current behavior, and how they define success and failure in sport. For Chinese collegiate athletes, who must manage academic responsibilities and sport training simultaneously, the ability to connect present training tasks with long-term development may be particularly important. The present study therefore extends procrastination research into the sport training context and provides a more specific psychological account of training engagement within the broader context of sustainable athlete development.

### 4.2 Future time perspective and training procrastination

The negative association between future time perspective and training procrastination supported H1. This finding is consistent with the theoretical view that future-oriented individuals are more likely to organize present behavior around long-term goals and consequences (Zimbardo and Boyd, 1999). In the context of sport training, many desirable outcomes, such as technical improvement, physical adaptation, tactical maturity, injury prevention, and competitive readiness, are not immediately visible. They usually depend on repeated effort and delayed returns. Therefore, athletes with a stronger future time perspective may be more likely to perceive daily training tasks as meaningful components of a longer developmental pathway.

This finding can also be interpreted through a sport time perspective framework. Stolarski et al. (2019) proposed that athletes’ temporal orientations may shape sport functioning through motivational engagement, affective regulation, performance appraisal, and coping with performance-related emotions. From this perspective, future time perspective may be relevant to training procrastination because training requires athletes to appraise current effort in relation to delayed sport outcomes, regulate discomfort during demanding practice, and interpret setbacks as part of long-term development rather than as reasons for avoidance. The present finding is consistent with this interpretation: athletes who more strongly connected present behavior with future outcomes tended to report less training procrastination.

This association is especially relevant for collegiate athletes. Unlike professional athletes whose institutional identity is mainly organized around sport performance, collegiate athletes often need to balance training with academic work, social life, and career preparation (Choi and Smith, 2024). In such a dual-role context, training tasks may compete with other immediate demands (García et al., 2023). When athletes have a clearer future orientation, present training may become more psychologically connected with future athletic development, personal growth, and role identity. This may explain why stronger future time perspective was associated with lower training procrastination in the present sample.

Within the context of sustainable athlete development, this result suggests that future time perspective may represent an important psychological correlate of consistent training engagement (Henry et al., 2017). Sustainable athlete development requires more than short-term performance improvement. It also involves continued participation, adaptive motivation, and the capacity to remain engaged in training over time. The present finding indicates that athletes’ orientation toward future consequences is closely linked to their tendency to delay or initiate training-related tasks (Husman et al., 2025). However, this association should not be interpreted as causal because of the cross-sectional design. It is also possible that athletes who experience less training procrastination develop a stronger sense of future direction through repeated successful training experiences. Therefore, the finding should be understood as evidence of a meaningful association rather than proof that future time perspective directly lowers training procrastination.

### 4.3 Self-control as a partial self-regulatory pathway

The mediation analysis showed that self-control statistically mediated the association between future time perspective and training procrastination, supporting H2. Athletes with stronger future time perspective tended to report higher self-control, and athletes with higher self-control tended to report lower training procrastination. This result is consistent with the view that procrastination is closely related to self-regulatory difficulty (Steel, 2007), while self-control reflects the capacity to regulate impulses, resist temptations, and act in accordance with long-term goals (Tangney et al., 2004). However, because the variables were assessed concurrently, this statistical mediation should not be interpreted as evidence that future time perspective temporally precedes self-control or that self-control causally transmits its association with training procrastination.

The partial mediation pattern is theoretically important. The indirect effect through self-control accounted for a relatively modest proportion of the total association between future time perspective and training procrastination. Therefore, self-control should be interpreted as one partial self-regulatory pathway rather than the sole mechanism linking future time perspective with training procrastination. Future-oriented thinking and self-control appear to be related but not interchangeable. Future time perspective may provide a broader temporal and motivational framework, whereas self-control may reflect a more immediate regulatory capacity involved in starting and maintaining training behaviors.

In the sport training context, this distinction is important. An athlete may understand that current training is important for future performance, but still experience difficulty initiating or completing training tasks when tired, distracted, emotionally unstable, or facing competing academic demands. Self-control may be especially relevant in these moments because it concerns the ability to act in line with longer-term intentions despite short-term discomfort or temptation (Duckworth and Gross, 2014). Thus, the present findings suggest that future-oriented thinking is linked to training procrastination partly through athletes’ self-regulatory capacity.

At the same time, the partial rather than full mediation indicates that other mechanisms may also be involved. Future time perspective may be associated with lower training procrastination through goal clarity, perceived training meaning, autonomous motivation, sport commitment, sport-specific self-efficacy, resilience, emotion regulation, habit strength, or planning behavior. From the perspective of Self-Determination Theory, athletes may be more likely to sustain effort when training goals are internalized, competence-supportive, and connected with personal development rather than experienced only as external demands (Ryan and Deci, 2000). Thus, future-oriented athletes may procrastinate less not only because they have stronger self-control, but also because they experience training as more meaningful, self-endorsed, and developmentally valuable. Future studies should examine these additional mediating mechanisms to build a more complete explanation of training procrastination in sport settings.

### 4.4 Mastery-approach goal orientation as a robust adaptive motivational boundary condition

The moderation result for mastery-approach goal orientation supported H3. In both the planned PROCESS model and the simultaneous-interaction robustness model, mastery-approach goal orientation strengthened the negative association between future time perspective and training procrastination. This suggests that the moderating role of mastery-approach goal orientation was not merely an artifact of omitting performance-avoidance goal orientation from the model.

This finding is theoretically coherent because mastery-approach goals emphasize personal improvement, skill mastery, effort, and performing as well as one possibly can (Conroy et al., 2003; Elliot and McGregor, 2001). These motivational features are highly compatible with future-oriented thinking. For athletes high in mastery-approach goal orientation, the future may be understood as a space for growth and development rather than merely as an external performance demand. Such athletes may be more likely to interpret daily training as a process of becoming better, refining skills, and approaching personal potential. Therefore, when future time perspective is accompanied by mastery-approach motivation, future-oriented thinking may be more strongly linked to lower training procrastination (Howell and Watson, 2007).

This finding also helps clarify why future time perspective may not operate in the same way for all athletes. A future-oriented athlete who lacks mastery-oriented motivation may still recognize the importance of future outcomes, but may not strongly associate current training tasks with self-improvement and skill development (Phan, 2009). By contrast, a future-oriented athlete with high mastery-approach orientation may view present training as a concrete pathway toward future progress. Thus, mastery-approach orientation may strengthen the practical meaning of future-oriented thinking in daily training.

Within the context of sustainable athlete development, this finding is particularly relevant. A mastery-approach orientation is consistent with long-term development because it directs athletes’ attention to controllable progress rather than only to external evaluation. In collegiate sport environments, where athletes may experience pressure from selection, rankings, competitions, and academic responsibilities, mastery-approach goals may help maintain a healthier motivational basis for training (Spray and Warburton, 2011). The present study suggests that the combination of future orientation and mastery-oriented motivation is especially relevant to lower training procrastination.

### 4.5 The qualified role of performance-avoidance goal orientation

The planned PROCESS model provided initial support for H4. In the separate moderation model, performance-avoidance goal orientation weakened the negative association between future time perspective and training procrastination. This pattern is theoretically understandable because performance-avoidance goals involve concern with avoiding poor performance, negative evaluation, or doing worse than others (Conroy et al., 2003; Elliot and McGregor, 2001). When athletes hold stronger performance-avoidance goals, future outcomes may be interpreted through a threat-based motivational frame. Instead of viewing the future as an opportunity for development, these athletes may associate the future with possible failure, negative evaluation, or unfavorable social comparison.

However, this moderation effect was attenuated in the simultaneous-interaction robustness model and became non-significant after mastery-approach goal orientation and the interaction between future time perspective and mastery-approach goal orientation were included. Therefore, the role of performance-avoidance goal orientation should be interpreted cautiously. The apparent moderating effect of performance-avoidance goal orientation in the separate model may partly reflect its overlap with lower mastery-approach orientation rather than a fully independent boundary condition. This interpretation is consistent with the moderate negative correlation between mastery-approach and performance-avoidance goal orientations observed in the present study.

This qualified pattern adds nuance to the role of future time perspective in sport. It is not enough to know whether athletes think about the future; it is also important to understand how they emotionally and motivationally interpret that future. For athletes high in performance-avoidance orientation, future-oriented thinking may sometimes be accompanied by pressure, defensive concern, or fear of negative comparison. When training tasks are difficult, evaluative, or likely to expose weaknesses, these athletes may experience future outcomes as threats rather than opportunities for growth (St-Cyr et al., 2023). Nevertheless, because the performance-avoidance interaction was not robust in the simultaneous model, this interpretation should be treated as a tentative explanation rather than a definitive conclusion.

The contrast between the robust mastery-approach effect and the qualified performance-avoidance effect also supports the idea that achievement goal orientations should not be treated as a single overall motivational score. Different goal orientations may shape the meaning of future-oriented thinking in qualitatively different ways. In the present study, mastery-approach orientation emerged as a more reliable motivational boundary condition, whereas the independent role of performance-avoidance orientation was more limited.

### 4.6 Cultural and educational context of Chinese collegiate athletes

The observed associations may reflect both general self-regulatory processes and context-specific features of Chinese collegiate sport. On the one hand, linking present training to future goals and regulating immediate impulses are likely to be relevant across athlete populations. Sport training in many contexts requires delayed gratification, repeated effort, tolerance of discomfort, and the ability to maintain engagement despite competing demands. Therefore, the associations among future time perspective, self-control, mastery-oriented motivation, and training procrastination may have relevance beyond the Chinese collegiate athlete population.

On the other hand, the Chinese collegiate sport context may shape how these psychological processes are expressed. Chinese collegiate athletes often train within educational institutions and face dual expectations for academic progression and sport performance (Lyu et al., 2022). This dual-role pressure may make future-oriented thinking especially salient because athletes need to connect daily training with both athletic development and broader educational or career pathways. At the same time, performance-avoidance concerns may be intensified in evaluative training environments where selection, ranking, competition opportunities, and coach feedback are visible.

This context may also help explain why mastery-approach goal orientation emerged as a robust boundary condition. For collegiate athletes who must coordinate academic and sport roles, mastery-oriented goals may help make training feel personally meaningful and developmentally constructive rather than merely another external demand. By contrast, when future goals are framed primarily around avoiding poor performance or negative comparison, the motivational value of future-oriented thinking may become less stable. Therefore, the present findings should be interpreted as reflecting both general psychological mechanisms and the specific educational-athletic environment in which Chinese collegiate athletes train.

### 4.7 Theoretical implications

This study offers several theoretical implications. First, it extends procrastination research into the sport training context. Previous procrastination research has mainly focused on academic, occupational, or general life domains. The present study examined training procrastination among collegiate athletes, thereby highlighting a form of procrastination that is embedded in long-term athletic development. Training procrastination is theoretically important because sport training is repetitive, physically demanding, and strongly future-oriented. By examining training procrastination as a sport-specific behavioral tendency, this study contributes to a more contextualized understanding of procrastination.

Second, this study contributes to the literature on time perspective in sport. Future time perspective has often been discussed as a general tendency to plan ahead and consider future consequences. The present findings suggest that, among collegiate athletes, future time perspective is meaningfully linked to training-related behavior and self-regulation. This aligns with the sport time perspective model proposed by Stolarski et al. (2019), which emphasizes that athletes’ temporal orientations may shape sport functioning through motivational, affective, and behavioral pathways. The present study extends this logic to training procrastination by showing that future-oriented thinking is associated with lower irrational delay in training.

Third, the study may inform future athlete-development research by positioning training procrastination as a meaningful behavioral tendency related to training consistency. Sustainable athlete development is often discussed in relation to health, wellbeing, motivation, and long-term participation (Seong, 2025). The present findings suggest that training procrastination may be one everyday behavioral pattern through which difficulties in self-regulation and motivation become visible. Frequent irrational delay in training may be associated with lower training consistency and less stable engagement. Therefore, training procrastination deserves more attention in athlete development research, especially among collegiate athletes who face both academic and sport-related demands.

Fourth, the study advances understanding of the process linking future time perspective with training behavior. The mediation result indicates that self-control statistically accounts for part of the association between future time perspective and training procrastination. This supports a process-oriented interpretation: future-oriented thinking is not only a cognitive orientation toward time, but is also linked to athletes’ capacity to regulate present behavior. However, because the indirect effect through self-control was partial, future research should examine additional psychological mechanisms such as autonomous motivation, sport-specific self-efficacy, perceived training meaning, resilience, and emotion regulation.

Fifth, the study contributes to achievement goal theory by showing that goal orientations function as motivational boundary conditions in the association between future time perspective and training procrastination (Elliot and Murayama, 2008). The robust role of mastery-approach goal orientation suggests that future-oriented thinking may be most strongly associated with lower training procrastination when future goals are embedded in self-improvement, learning, and controllable progress. The less robust role of performance-avoidance goal orientation further suggests that avoidance-based motivation may be more difficult to interpret independently, especially when it overlaps with lower mastery-oriented motivation.

Finally, the study provides evidence from Chinese collegiate athletes, a population that is theoretically and practically important. Chinese collegiate athletes often train within educational institutions and must coordinate academic and athletic responsibilities. Their development cannot be understood only through performance outcomes or training volume. It also requires attention to psychological regulation, motivational orientation, and sustained engagement. By focusing on this group, the present study helps situate training procrastination and sustainable athlete development within a specific educational and cultural context.

### 4.8 Practical implications

Because the present study was cross-sectional and did not evaluate an intervention, the following implications should be interpreted as provisional considerations for coach education, athlete support, and future intervention research rather than as tested intervention effects. The findings provide several practical implications for coaches, sport educators, university athletic departments, and psychological support staff working with collegiate athletes. First, training procrastination should be treated as more than a simple discipline or time-management issue. The present findings suggest that training procrastination is associated with future time perspective, self-control, and achievement goal orientations. Therefore, when athletes frequently delay training tasks, coaches and educators may need to consider whether the athlete has a clear sense of future development, sufficient self-regulatory strategies, and an adaptive motivational orientation toward training (Schmidt et al., 2009).

Second, athlete development programs may benefit from helping collegiate athletes build a clearer and more concrete future time perspective. This should not be limited to abstract long-term goal setting. Athletes may need guidance in connecting specific daily training tasks with future outcomes. For example, strength training can be linked to future injury prevention and competition readiness, technical drills can be linked to long-term skill refinement, and recovery routines can be linked to training continuity and health. Such future-oriented reflection may help athletes perceive training tasks as meaningful parts of a developmental pathway rather than isolated obligations (Barkoukis et al., 2010).

Third, self-control should be addressed through practical self-regulation strategies. Since self-control partially mediated the association between future time perspective and training procrastination, athlete-support programs may need to address both motivation and self-regulatory execution. For collegiate athletes, this may include planning training around academic schedules, using implementation intentions, establishing pre-training routines, setting short-term process goals, reducing mobile phone distractions before training, and using self-monitoring logs to track training completion. Implementation intentions may be especially useful because they help individuals link intended actions to specific situational cues, thereby supporting goal-directed behavior (Gollwitzer, 1999).

Fourth, coaches should cultivate mastery-approach climates. The finding regarding mastery-approach goal orientation suggests that future-oriented thinking is more strongly linked to lower training procrastination when athletes define success in terms of personal progress and skill mastery. Coaches may support this by emphasizing controllable improvement, effort quality, technical refinement, and individual progress. Feedback should not focus exclusively on comparison, selection, or rankings. Instead, coaches can help athletes identify what improved, what needs adjustment, and how current training connects to long-term development. Such a climate may support healthier and more sustainable training engagement (Adie et al., 2008).

Fifth, practitioners should be cautious about excessive performance-avoidance framing. The separate moderation model suggested that performance-avoidance goal orientation may weaken the negative association between future time perspective and training procrastination, although this pattern was not robust in the simultaneous-interaction model. Therefore, messages that overemphasize failure, ranking loss, or negative comparison should be used carefully, especially for athletes who already show strong avoidance concerns. For these athletes, coaches and psychological support staff may consider reframing future goals in terms of growth and controllable progress rather than threat avoidance (Stoeber et al., 2008).

To make these implications more concrete, Table 11 summarizes coach-oriented strategies that may be considered according to different motivational and self-regulatory profiles. These strategies are intended as practical applications derived from the present findings rather than as tested intervention effects.

**TABLE 11:** Illustrative coach-oriented considerations for addressing training procrastination according to motivational and self-regulatory profile.

Athlete profile	Main risk	Feedback focus	Coach-oriented strategies
Low future time perspective	Weak link between current training and future goals	Connect daily training with long-term development	Use season goal maps; divide long-term goals into weekly targets; explain how specific drills, recovery routines, and strength training support future performance
Low self-control	Difficulty initiating or persisting in planned training	Reduce starting barriers and strengthen action cues	Use implementation intentions; establish pre-training routines; schedule training around academic demands; manage phone distraction; use training completion logs
High mastery-approach goal orientation	Adaptive profile that can support sustained engagement	Reinforce progress, effort, and skill mastery	Use process goals; provide technical feedback; track personal-best indicators; review progress across training cycles
High performance-avoidance goal orientation	Tendency to interpret training and evaluation as threats	Reduce shame-based comparison and failure-focused feedback	Provide private corrective feedback; normalize mistakes; emphasize controllable improvement rather than ranking loss
High future time perspective + high performance-avoidance goal orientation	Future awareness accompanied by evaluation anxiety	Reframe goals from avoiding failure to achieving progress	Use goal-rewriting exercises; combine future planning with emotion regulation; convert outcome threats into controllable process targets
Low mastery-approach goal orientation + high training procrastination	Weak mastery meaning and low perceived training value	Increase task value and perceived competence	Use small achievable goals; provide immediate feedback; record skill mastery; encourage peer support and coach-guided reflection

The strategies are practice-oriented applications of the present findings and should be interpreted as suggestions for coach education and athlete support rather than as evidence of tested intervention effects.

### 4.9 Limitations and future directions

Several limitations should be acknowledged. First, the study used a cross-sectional design. Although the statistical model was theoretically grounded, the results cannot establish causal or temporal relationships among future time perspective, self-control, achievement goal orientations, and training procrastination. Future research should use longitudinal designs to examine whether these variables change together across a semester, a training cycle, or a competition season. Such designs would help clarify whether future time perspective precedes changes in training procrastination, whether self-control changes over time, and whether achievement goal orientations alter the strength of these associations across different training stages.

Second, all variables were measured using self-report questionnaires. Although the common method bias analyses argued against a dominant single-factor explanation of the covariance structure, they cannot rule out other forms of common-method variance. Self-report data may still be influenced by social desirability, memory bias, or participants’ subjective interpretation of training procrastination. Future research could combine self-report measures with behavioral indicators, such as training attendance records, completion of planned training tasks, coach ratings, training logs, or digital training platform records. Such multi-source data would provide a more detailed understanding of training procrastination.

Third, the study adapted the Irrational Procrastination Scale to the sport training context. Although the adapted scale showed strong internal consistency and preliminary psychometric support in the present sample, this should not be interpreted as complete validation of a sport-specific training procrastination measure. Training procrastination may involve sport-specific behaviors that are not fully captured by a general procrastination scale. For example, athletes may delay rehabilitation exercises, recovery routines, technical correction, video analysis, strength training, or communication with coaches. Future studies could develop and validate a dedicated training procrastination scale that reflects these sport-specific forms of irrational delay.

Fourth, the present study focused only on the Future subscale of the Zimbardo Time Perspective Inventory. Other time perspective dimensions, particularly present-hedonistic and present-fatalistic orientations, were not measured. These dimensions may be relevant to impulsive behavior, avoidance, and procrastination. Future studies should examine whether present-hedonistic and present-fatalistic orientations show independent or interactive associations with training procrastination.

Fifth, the present study focused on mastery-approach and performance-avoidance goal orientations as two theoretically contrasting moderators. This decision allowed for a clearer and more parsimonious model, but it also means that mastery-avoidance and performance-approach goal orientations were not included in the main moderated mediation analysis. These two orientations may have more complex roles. Performance-approach goals may be associated with stronger engagement in competitive settings, but may also be linked to pressure when social comparison becomes excessive. Mastery-avoidance goals may reflect high personal standards, but may also involve concern about not reaching one’s own potential. Future research could examine the full 2 × 2 achievement goal framework to determine whether different goal orientations show distinct conditional associations with training procrastination.

Sixth, training years and sport history were treated as background or control variables rather than examined as focal predictors or moderators. Future studies could test whether the psychological pathways identified in this study differ across levels of training experience, athletic level, and competition history. Such analyses may clarify whether future time perspective, self-control, and achievement goal orientations operate similarly for early-stage collegiate athletes and more experienced athletes.

Seventh, the study used a multi-institutional convenience and network-based sampling strategy. Although participants were recruited from multiple universities and regions, athletes who were more available, more motivated, or more interested in psychological topics may have been more likely to participate. This self-selection bias may limit the generalizability of the findings. In addition, specific sport disciplines were not collected; therefore, sample heterogeneity could only be examined at the broader individual/team sport level. Future research should collect more detailed sport-discipline information to examine whether training procrastination differs across sports with different training structures, evaluation systems, and seasonal demands.

Eighth, although several contextual variables were collected, subgroup and multigroup analyses were not conducted in the present study. Future research should test whether the proposed model is invariant across gender, sport type, athletic level, training years, competition level, and injury status. Such analyses may provide a more nuanced understanding of whether the psychological processes associated with training procrastination are consistent across different athlete subgroups.

Ninth, self-control was measured as a general trait. Although general self-control was meaningfully associated with training procrastination, sport-specific self-control may provide additional explanatory value. Some athletes may show strong self-control in academic or daily life contexts but still struggle with training-specific avoidance. Future studies could examine sport-specific self-control, training planning ability, habit strength, and emotion regulation as additional psychological variables. It would also be useful to test whether training-specific self-regulation strategies are associated with changes in training procrastination over time.

Finally, although the study was framed within the context of sustainable athlete development, broader developmental outcomes were not directly measured. These outcomes may include psychological wellbeing, burnout, injury risk, academic adaptation, sport commitment, and long-term participation intention. Therefore, the findings should not be interpreted as direct evidence of broader athlete sustainability outcomes. Future research could examine whether training procrastination is associated with these outcomes and whether future time perspective, self-control, and achievement goal orientations show indirect or conditional associations with broader indicators of athlete health, welfare, and sustainable development.

## 5 Conclusion

This study examined training procrastination among Chinese collegiate athletes by integrating future time perspective, self-control, and achievement goal orientations. Future time perspective was associated with lower training procrastination, and this association was partially accounted for by self-control. Mastery-approach goal orientation robustly strengthened the negative association between future time perspective and training procrastination, whereas performance-avoidance goal orientation showed a weakening pattern in the separate moderation model but was not robust in the simultaneous-interaction model.

These findings suggest that training procrastination is related not only to athletes’ future-oriented thinking and self-regulatory capacity, but also to the motivational meaning they attach to training and performance. Within the context of sustainable athlete development, the results may inform coach education and athlete-support practices that link daily training with future goals, support self-regulation, and encourage mastery-oriented feedback. These findings should be interpreted as cross-sectional associations and do not directly demonstrate broader sustainable athlete development outcomes or intervention effectiveness. Given the cross-sectional design, longitudinal and intervention studies are needed to clarify causal and developmental processes.

## Data Availability

The datasets presented in this study can be found in online repositories. The names of the repository/repositories and accession number(s) can be found at: “The data supporting the findings of this study are openly available in Zenodo at https://doi.org/10.5281/zenodo.20042927.”
