# Supplementary figures and images for: Future time perspective and training procrastination in Chinese collegiate athletes: self-control mediation and the roles of mastery-approach and performance-avoidance goals
Source: Front Psychol. 2026 Jul 15;17:1873288. doi: 10.3389/fpsyg.2026.1873288 (PMC13415922; doi:10.3389/fpsyg.2026.1873288)

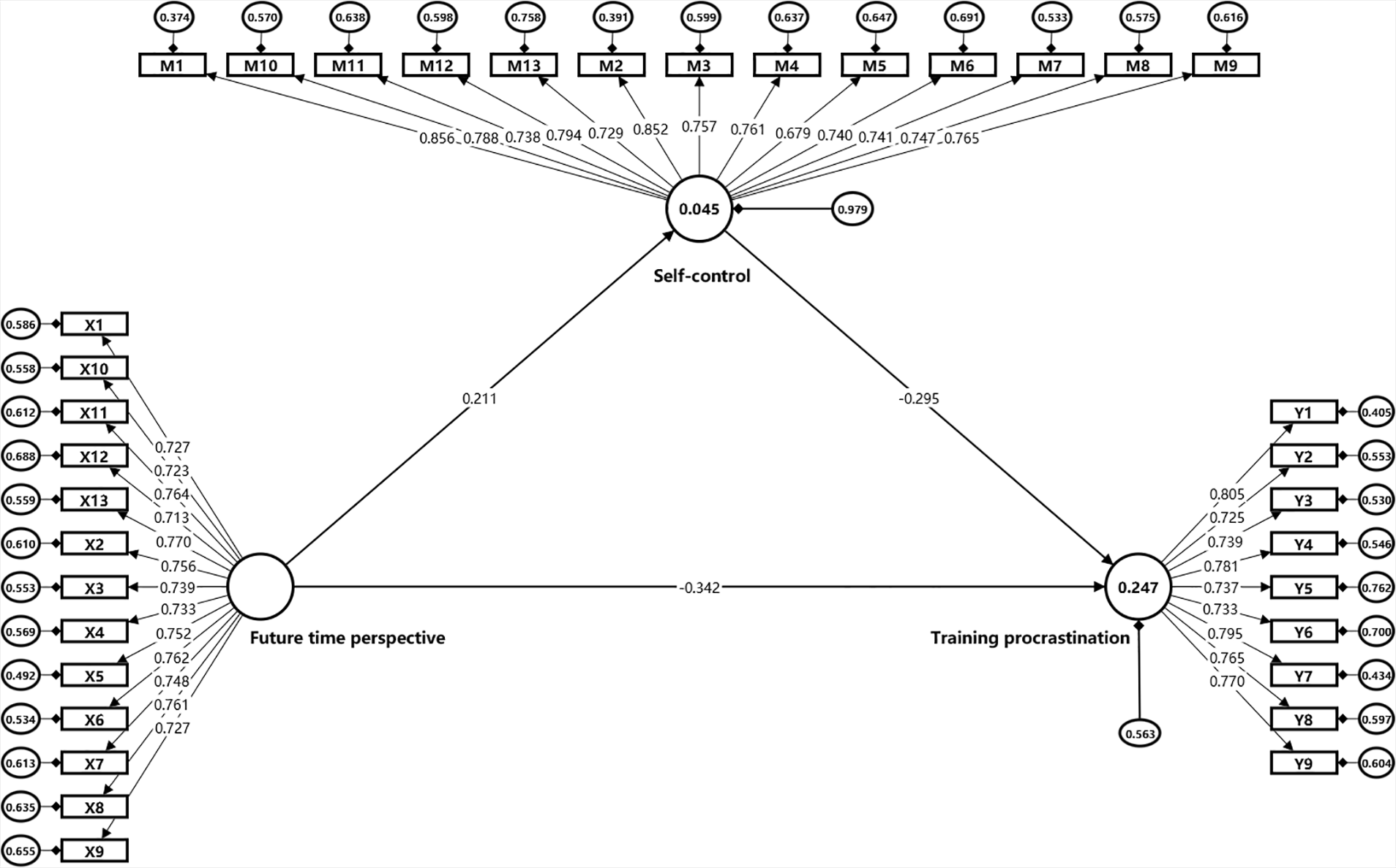

Supplement: Supplementary file 1 [file Image_1.tiff]
